# High-Throughput Functional Screening of Steroid Substrates with Wild-Type and Chimeric P450 Enzymes

**DOI:** 10.1155/2014/764102

**Published:** 2014-08-26

**Authors:** Philippe Urban, Gilles Truan, Denis Pompon

**Affiliations:** ^1^Université de Toulouse, INSA, UPS, INP, LISBP, 135 Avenue de Rangueil, 31077 Toulouse, France; ^2^INRA, UMR792 Ingénierie des Systèmes Biologiques et des Procédés, 31400 Toulouse, France; ^3^CNRS, UMR 5504, 135 Avenue de Rangueil, 31400 Toulouse, France

## Abstract

The promiscuity of a collection of enzymes consisting of 31 wild-type and synthetic variants of CYP1A enzymes was evaluated using a series of 14 steroids and 2 steroid-like chemicals, namely, nootkatone, a terpenoid, and mifepristone, a drug. For each enzyme-substrate couple, the initial steady-state velocity of metabolite formation was determined at a substrate saturating concentration. For that, a high-throughput approach was designed involving automatized incubations in 96-well microplate with sixteen 6-point kinetics per microplate and data acquisition using LC/MS system accepting 96-well microplate for injections. The resulting dataset was used for multivariate statistics aimed at sorting out the correlations existing between tested enzyme variants and ability to metabolize steroid substrates. Functional classifications of both CYP1A enzyme variants and steroid substrate structures were obtained allowing the delineation of global structural features for both substrate recognition and regioselectivity of oxidation.

## 1. Introduction

In mammals, P450 enzymes of hepatocyte endoplasmic reticulum play a major role in the oxidative metabolism not only of xenobiotics (drugs, environmental pollutants, and phytochemicals) [1–4] but also of endogenous compounds such as steroids [5]. It has been shown that, similarly to drug chemicals, steroids are frequently transformed into several metabolites by P450 enzymes involved in drug metabolism [6, 7]. Progesterone is hydroxylated at the 6 and 21 positions by human CYP2C9 in addition to carbon-16 for human CYP3A4 and 16 and 17 for human CYP2C19 [8]. However, in contrast to the wide variety of drug shapes, steroids present a common chemical androstane skeleton which limits the structural diversity of the nature of substituting groups at the different available position of the androstane ring as shown in Figure 7.

The particular diversity of steroids therefore appears particularly adapted to explore the structural factors that control enzyme regiospecificity of action and, reciprocally, to identify the general features of steroid structures which control recognition by P450 enzymes.

We focused this work on natural and synthetic CYP1A enzymes and a collection of 16 steroidal substrates, 14 steroids, and 2 steroid analogues, a terpenoid and a drug. The effect of the diversity of steroid chemical structures was assessed by using different substrates of increasing complexity in their substituent groups. The effect of the diversity of P450 enzyme structures was assessed by using a library of artificial chimeric CYP1A enzymes of increasing shuffling complexity as described previously [9]. The purpose of this study was to assess to what extent a statistical approach relying on kinetics could be used to identify and predict global structural determinants of substrate-enzyme recognition. Interestingly, the X-ray structural information now available for CYP1A P450 proteins [10, 11] does not evidence any clear features explaining how major differences of activities among CYP1A enzymes could be related to local structural differences. Statistical approaches thus constitute an alternative but they require sufficiently large datasets of experiments thus relying on high-throughput methods. The steady-state rates were determined by monitoring metabolites produced from a given steroid substrate by LC/MS following different incubation times in 96-well microplate using yeast-expressed recombinant mammalian CYP1A enzyme. Due to the large number of enzyme-substrate couples, each giving frequently several metabolites, only measurements at saturating substrate concentrations were performed.

The choice to study CYP1A enzymes instead of CYP2 or CYP3 enzymes, which are well known to oxidize steroids, is mainly due to the fact that CYP1A enzymes are less numerous than CYP2C ones and, thus, more easily amenable to interpretable combinatorial studies. Moreover, CYP1A enzymes do not exhibit cooperativity in their kinetics contrary to what is observed with CYP3A enzymes. In these respects, CYP1A enzymes are simpler and, therefore, better adapted to be used as models of steroid oxidation, as is the case in this work.

## 2. Materials and Methods

### 2.1. Substrates

The steroid series comprises testosterone, 17-methyltestosterone, progesterone, pregnenolone, estrone, cortisol, 19-norandrostenedione, dehydroepiandrosterone (DHEA), cortexolone, corticosterone, 17-hydroxy- and 21-hydroxyprogesterone,* cis*-androsterone (3α-hydroxy-5α-androstan-17-one),* trans*-androsterone (3*β*-hydroxy-5α-androstan-17-one), and two steroid analogs: nootkatone and mifepristone (RU486). Mifepristone was a gift from Roussel-Uclaf (Romainville, France). All other steroids were from Sigma. The different substrates were solubilized either in methanol (since ethanol is a known CYP1A inhibitor) or in dimethylformamide (DMF).

### 2.2. Plasmids and Yeast Strain

Vectors p1A1/V60 and p1A2/V60 for human wt CYP1A expression, pP1V8 for mouse wt 1A1 expression, and pLM4V8 for rabbit wt 1A2 expression were described before [12, 13]. The pYeDP60 vector contains both* URA3* and* ADE2* as selection markers, whereas pYeDP8 only bears* URA3*. The inserted coding sequence is placed under the transcriptional control of a* GAL10*-*CYC1* hybrid artificial promoter and* PGK* terminator.

*Saccharomyces cerevisiae* W(R) strain is a derivative of the W303-1B which, when cultivated on galactose, overexpresses yeast NADPH-P450 reductase. For expression of P450 enzymes, the W(R) yeast strain was chosen since yeast NADPH-P450 reductase overexpression optimizes the activities of any recombinant P450. After transformation and growth on selective medium, positive clones were selected for mitochondrial respiration on plates containing glycerol [14]. Several well-growing clones were used to inoculate 50 mL of SGI selective liquid media. This culture was grown overnight to reach stationary phase and was used to inoculate 250 mL of YPGE liquid culture. After 48 hours growing on constant shaking and at 28°C, 5 g of galactose was added to the culture for the overnight induction of the expression of both the cloned gene and the yeast P450 reductase.

### 2.3. Microsomal Fractions Preparation

Briefly, yeast cells were harvested by centrifugation, suspended, and washed in 50 mM Tris-HCl 1 mM EDTA 0.6 M Sorbitol (TES) buffer pH7.3. Cells were disrupted by manual shaking with 0.4 mm diameter glass beads. Cellular debris were removed by centrifugation (10 min at 10,000 rpm). The supernatant was transferred to another centrifuge tube, and NaCl and PEG4000 were added at final concentrations of 0.1 M and 10%, respectively, and kept on ice for 30 minutes. The precipitated microsomes were then pelleted by centrifugation 10 min at 10,000 rpm, washed, and resuspended in 50 mM Tris-HCl 1 mM EDTA 20% glycerol, pH7.4 buffer, and stored in −80°C [14].

### 2.4. Chimeric CYP1A Enzyme Variants

Four parental P450s were used in DNA shuffling experiments: human CYP1A1 and CYP1A2 mouse CYP1A1 and rabbit CYP1A2 coding sequences. Two shuffling methods have been applied to build a library of increasing complexity ranging from bi- or tripartite chimera up to highly mosaic structures (average 5-6 crossovers per sequence). The bi- or tripartite chimeras were obtained by* in vivo* gap-repair technology [15] between mouse CYP1A1 and rabbit CYP1A. The mosaic CYP1A variants were obtained using the mixed* in vivo, in vitro* CLERY recombination procedure between human CYP1A1 and CYP1A2 [16]. These two libraries of chimeric sequences coding for functional variant mammalian CYP1A enzymes have been previously described [9].

### 2.5. Enzyme Activities

Incubations with steroids (initiated by NADPH addition), reaction quenching with trifluoroacetic acid (2 : 250 by vol.), and acetonitrile extractions were automatically performed with a QIAgen 8000 robot using a homemade QIAsoft program. The high-throughput procedure consists in incubation of the different enzyme, substrate pairs in each well of a 96-well microplate. Microsomal fractions of recombinant yeast clones each expressing a particular CYP1A enzyme were assayed with the 16 steroid substrates in a round-bottom 96-well microplate (well volume = 0.30 mL) by incubation at 30°C for 0, 5, 10, 15, and 20 min. Incubation mixtures contained 0.3–0.6mg of microsomal yeast proteins and their concentration in recombinant P450 was ranging from about 5 up to 30 nM (as assessed by CO-reduced differential spectrum on some preparations). These variations were eliminated by the statistical treatment described further. A systematic control was included for each chemical tested and consisted of 20 min incubation with microsomal fractions prepared from W(R) cells transformed by void vector. After incubation completion, microplate content was transferred by the robot to a second microplate for acetonitrile treatment (1 : 1 by vol.) and, after centrifugation (5 min at 3,500 rpm), the 96 supernatants of this microplate were transferred to a third 96-well Porvair microplate which fits in the automatic injector compartment of an Alliance HT2795 HPLC Waters module. For all steroid substrates, the initial concentration in incubation mixtures was 100 *μ*M, a value that generally corresponds to enzyme saturation while remaining below the solubility limits. Microsomal steroid hydroxylation and mifepristone N-demethylation activities were shown to be strictly NADPH-dependent.

### 2.6. Analytical LC/MS Methods

The acetonitrile supernatants were LC-separated at 40°C with an XTerraMS C_18_ 5 *μ*M 4.6 × 100 mm column (Waters) and analyzed on a Micromass ZQ single quadrupole mass spectrometer (Waters). The solvent system consisted of H_2_O + 0.01% formic acid (by vol.) in acetonitrile + 0.01% formic acid (by vol.). The gradient used (flow rate of 1.0 mL/min) for all steroid metabolites starts at 90 : 10 (water : acetonitrile), followed by linear increase from 85 : 15 to 0 : 100 over 8 min, return to initial conditions and hold for 2 min; 10 min of total run length. Parameters of the electrospray positive ionization were as follows: capillary voltage 3.4 kV, cone voltage 20.0 V, desolvation gas flow  550 L · h^−1^, desolvation temperature 350°C, and source temperature 120°C. Continuous metabolite mass detection was using both full scan spectra by scanning mass range 200–500 amu and several SIR channels (single ion response) set at the precise *m*/*z* corresponding to the expected protonated metabolites (hydroxylated derivatives of all substrates plus the N-demethylated metabolite of mifepristone). With the masses of each substrate being known, it was possible to specifically detect the mass of predicted hydroxylated (+16 amu) and N-demethylated products (−14 amu). The detected *m*/*z* corresponds to [M+H]+ since positive electrospray mode is used. Metabolites were quantified by measuring peak areas on mass spectra chromatograms. The area of each MS detected product peak was plotted versus the time of incubation (0, 5, 10, 15, and 20 min) yielding the rate of reaction. MS response coefficients are linked to ionization efficiencies and can differ between metabolites. In the absence of suitable standards for all metabolites, absolute calibration cannot be performed but due to the similar ionization mechanisms for all ketosteroids, the observed signal was considered to correlate relative metabolite amounts. However, a suitable normalization procedure was applied for statistical analysis.

### 2.7. Statistics

A normalization procedure based on the variance in the dataset was used as previously described [9]. CYP1A activities for the different steroidal substrates were analyzed using principal component analysis (PCA) and multidimensional scaling (MDS). PCA visualizes systematic patterns or trends of variation in large data set. The different trends of variation hidden in the initial multidimensional space are evidenced since the new orthogonal axes of the projected space (the two first principal components) are derived from the directions of larger variability [17]. MDS is a nonlinear projection of the distances separating each object from the others in the original multidimensional space into a 2- or 3-dimensional diagram designated as the MDS configuration plot [18]. MDS enables one to easily visualize and evaluate distances between two objects considering at the same time the influence of all other objects. An indicator to evaluate the quality of a MDS analysis is calculation of a diagnostic index known as Kruskal's stress which varies from 0 (a perfect fitting) up to 1 (no fitting). It measures the closeness of the distance mapping in the 2D MDS plot compared to the “real” distances in the original space. The multivariate statistics and dendrogram construction were performed by using Addinsoft XLSTAT software. Datasets and correlation matrices used throughout this work are available upon email request from urban@insa-toulouse.fr.

### 2.8. Human CYP1A Structures

The atomic coordinates were taken from the Protein Data Bank, RCSB, Rutgers University (entry: 4i8v for human CYP1A1 at 2.6 Å resolution and 2hi4 for human CYP1A2 at 1.95 Å resolution) [10, 11]. Protein visualizations were performed by using PyMol (Delano Scientific LLC, San Carlos, CA, USA).

## 3. Results and Discussion

### 3.1. Experimental Design for Activity Matrix Acquisition

This work is a combinatorial approach of protein quantitative structure-activity relationships (QSAR) [19]. A large collection of chimeric enzymes (all related by sharing a high degree of sequence similarity due to the fact that the parental enzymes are homologous) was prepared by shuffling sequence elements between two parental wild-type enzymes [9].

The library of chimeric structures combining members of the CYP1A subfamily was built starting from four parental wild-type enzymes, two CYP1A1s and two CYP1A2s, originating from different mammals (human, mouse, and rabbit). Human CYP1A1 and CYP1A2 amino acid sequences present a 73% identity, placing them over the limits for effective recombination using annealing-based methods. In our case, these two sequences were shuffled using the CLERY method [16]. This shuffling produced mosaic sequences with an average of 5 crossovers per sequence. The expressed functional CYP1A mosaic variants are collectively designated as ChiMo enzymes and numerated individually from ChiMo1 up to ChiMo11. The second library of chimera was constructed by shuffling sequences between mouse CYP1A1 and rabbit CYP1A2. Their 65% amino acid identity places them below the limits for effective PCR-based recombination techniques. The chimeras were obtained by using an* in vivo* technique based on gap repair in yeast [15]. This shuffling produced bi- or tripartite chimeric sequence. The expressed functional CYP1A chimeric variants are collectively designated as Chim enzymes and numerated individually from Chim3 up to Chim14.

**Figure 1:**
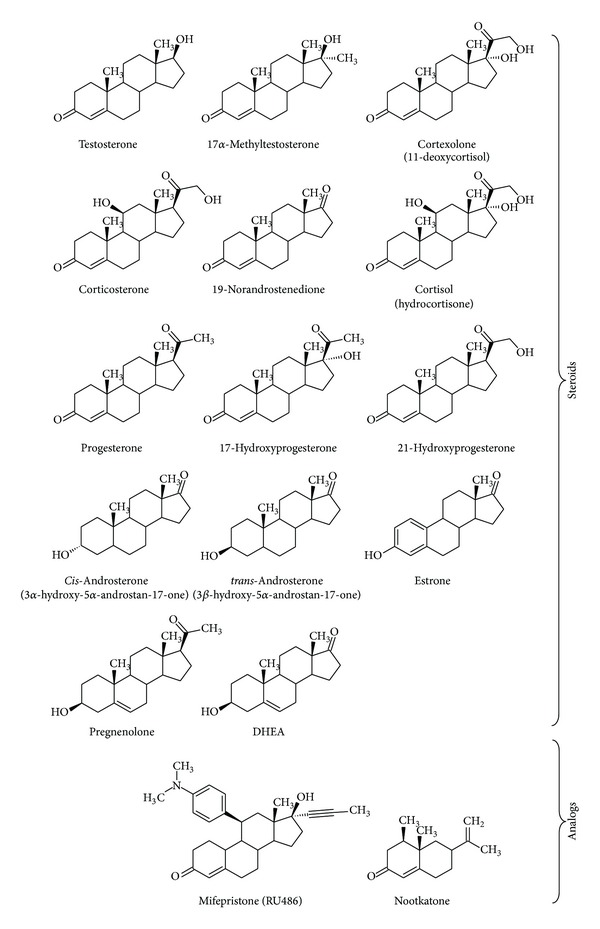
Chemical structures of the steroids and steroid analogues used in this work as substrates to assay CYP1A enzymes.

**Figure 2:**
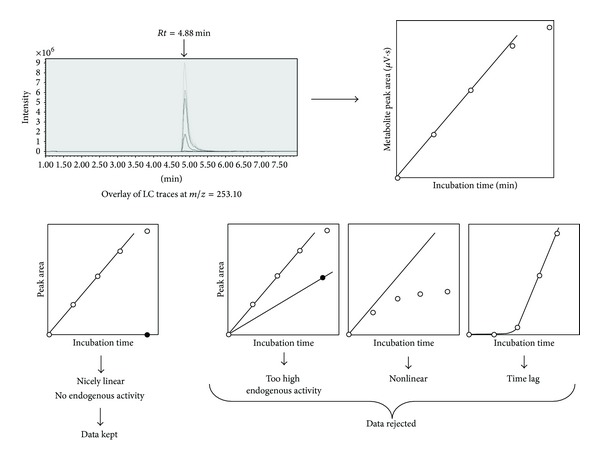
Flowchart for HT activity analysis. Upper left panel, LC/MS screenshot of the overlay of six chromatograms observed for CYP1A1-catalyzed hydroxylation of a substrate of *m*/*z* = 237 at different incubation times (hydroxylated substrate [M + H] + = 253). Upper right panel, area of the metabolite peak at *Rt* = 4.88 min versus incubation time. The four lower panels present different types of observed kinetics and rejection cases. Left panel, the case is kept for further analysis (metabolite production is linear over time).

We assayed the wild-type and chimeric P450 enzymes (*n* = 31) with a collection of 16 steroids and 2 analogs (Figure 1). Mifepristone is an N-dimethylated steroid analog and both hydroxylation reactions and N-demethylation reactions can be observed with CYP1A enzymes. All other substrates were monitored for CYP1A-catalyzed hydroxylase activity. With this set of substrates, 82 different metabolites were detected by mass spectrometry after separation of the incubation mixture by reverse-phase LC. Each of these metabolites specifies an activity; the profile of all detected metabolites produced from a single steroid substrate allows accessing a regiospecificity index (to be defined latter) for the action of a particular CYP1A enzyme. For each pair (enzyme, activity), the initial rates of metabolite formations were measured using the same substrate concentration (100 *μ*M) chosen to be generally saturating and within the range of solubility of the whole set of chemicals. Experimentally the 31 different enzymes and 18 substrates represent 558 (enzyme, steroid) pairs, leading to 3348 individual incubations considering that a 5-point time series (0, 5, 10, 15, and 20 min) and a negative control (void expression vector and 20 min incubation time) were performed for each condition (see Table S1 in Supplementary Material available online at http://dx.doi.org/10.1155/2014/764102). Assays were duplicated using at least two independent microsomal preparations and hidden replicates were included. The initial rates determination was achieved in a reasonable amount of time with the help of high-throughput technology. Only activity-enzyme pairs for which the time course of metabolite accumulation appeared to be linear with a negligible background endogenous activity were further considered for analysis (Figure 2).

### 3.2. Data Normalization for Analysis

Since kinetic analyses were not carried out with purified enzymes and because P450 contents in microsomal fractions were not systematically determined due to limited sensitivity of spectral quantification with certain microsomal fractions, actual catalyst contents from a yeast microsomal preparation to the other were not systematically assessed. Two parallel normalization treatments were thus carried out on the raw activity data.

The first treatment of dataset consisted in unit scaling of activities; it is aimed to easily visualize the full dataset by normalizing the activities of all metabolites in a way that each activity value ranges from 0 to 1. This makes a direct comparison of the dataset possible even if the raw unprocessed values widely differ, as is the case here. To do so, for each metabolite, the highest signal observed is arbitrarily set at 1. All other activities determined for this metabolite with all other enzymes are expressed as a ratio of their values to the highest activity observed. This makes all activity values for each metabolite to range from 0 to 1 after this data processing. Such a processing was already used for activity data visualization purposes by others [20].

The second treatment of dataset consisted in a normalization based on the variance in the dataset as described previously [9]. This variance normalization [21] eliminates the arbitrary factor linked to differences in expression levels of enzyme variants. A possible drawback could be that global substrate selectivity features of the whole enzyme collection are not corrected and could introduce some arbitrary weighting of data. Two dimensionality reduction statistical approaches: principal component analysis (PCA) and multidimensional scaling (MDS), were performed on the variance-normalized dataset to obtain projections of the data in two-dimensional diagrams. The two statistical approaches, PCA and MDS, are complementary and somewhat slightly affected by unavoidable data bias. PCA is a linear approach fairly sensitive to weighting and by the way the “distances” between activities are calculated (the metrics). MDS is in contrast a nonlinear projection, frequently less sensitive to these factors which, in contrast to PCA, do not necessarily yield a unique solution.

### 3.3. Structural Features Important for a Steroid to Be a CYP1A Substrate

Correlations existing between substrate structural elements and the activities were first investigated using a subset of the dataset for the sake of clarity. This subset includes only the wild-type CYP1A enzymes and the activities corresponding to the principal metabolite produced for each steroidal substrate. The analysis was limited to wild-type enzymes since the process of chimera formation by sequence shuffling may alter the typical profile of the protein structural elements that fit the substrate compared to the original profile in the wild-type enzyme. Similarly, the analysis was limited to the main activities for each steroidal substrate because the main product being produced at a high rate implies an optimal fitting (in the general acceptance of this term) of the substrate molecule within the catalytic cavity of the enzyme.

**Figure 3:**
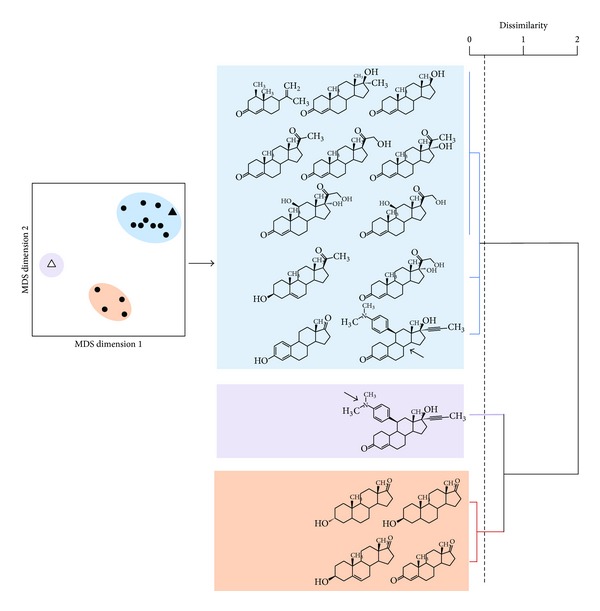
Visualization of how wild-type CYP1A enzymes differentiate steroidal substrates. Left panel, MDS configuration plot based on the ratio model and characterized by a stress index of 0.016. Each point represents the main activity observed for each one of the steroidal substrates tested in this work except for mifepristone which is represented in this plot by two points (triangles). The solid triangle represents the main mifepristone (RU-486) hydroxylase activity with all CYP1A enzymes; the open triangle represents mifepristone N-demethylase activity. Right panel, dendrogram deduced from the correlation matrix used to build the MDS configuration plot. The dendrogram was built by the Ward method based on the dissimilarity matrix.

The correlation matrix (i.e., dissimilarity matrix) was deduced from this subset and used to extract a MDS configuration plot in which the objects shown are steroid substrates scattered throughout the plot depending on their behavior towards wild-type CYP1A enzymes taken globally (Figure 3, left panel). Two steroid substrates will be found close together in this MDS configuration plot if and only if their behaviors toward the considered set of wild-type CYP1A enzymes are similar; the closer, the more similar. On the contrary, steroid substrates which are dissimilar for the CYP1A enzymes are found to be plotted far from each other.

Activities toward steroid substrates fall within three distinct groups, one of them involving two subgroups. The mifepristone N-demethylase activity appears at a well-separated position on the graph. A surprising observation is that the same substrate belongs to a distantly related group when steroid hydroxylation is considered. A likely explanation of this fact would be that the N-demethylation reaction takes place at a side of the mifepristone molecule opposite to the side at which hydroxylation reactions occur. This strongly suggests that hydroxylation regioselectivity plays an important role in the graph structure, hence in CYP1A metabolism of steroids.

A second group contains four steroids, two androsterones (*cis* and* trans*), DHEA, and 19-norandrostenedione. The fact that both* cis*- and *trans*-androsterones (resp., 3α-hydroxy-5α-androstan-17-one and 3*β*-hydroxy-5α-androstan-17-one) both fall in the same group suggests that orientation of the hydroxyl-group at position 3 of the steroid molecule is not determining for steroid recognition by CYP1A enzymes. All these steroids are characterized by no or small side groups on their molecular scaffold, particularly at C17 position which is always a keto group in this group. However, this feature is not fully characteristic of the group and a contrasting example can be found in the third group.

This third group contains most of the steroid substrates, including the mifepristone hydroxylase activity. Most of these steroids present two bulky groups at the C-17 position. Estrone, which is also a simple steroid molecule, falls in this group but its *A* ring is aromatic contrary to all other steroidal substrates tested. Due to *A* ring aromaticity, estrone is more testosterone-like and this could explain its classification in this third group. The terpene nootkatone, a steroid analog, is also found in this third group and not as an outlier, as is observed for mifepristone.

A dendrogram built by the Ward method calculated from the correlation matrix used for MDS analysis is shown in Figure 3, right panel. The chemical structure of corresponding steroid substrate is indicated in front of each branch of the resulting tree. This helps visualizing the main structural determinants on steroidal substrates that are differentiated by wild-type CYP1A enzymes and, therefore, are critical for a steroid molecule to be a substrate well recognized by CYP1A enzymes.

**Figure 4:**
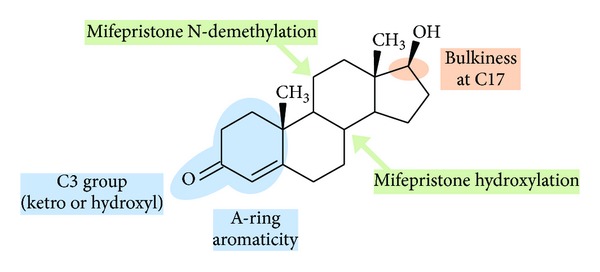
Features that were found important for efficient recognition of a steroidal substrate by CYP1A enzymes.

A closer examination of the large upper group on the dendrogram illustrates that this group could be split into several subgroups since the main branch of the dendrogram gives rise to three individual branches of low dissimilarity. All steroidal substrates found in the upper branch have in common to present both a bulky group found at C17 position and a keto group at C3 with a 4-androstene skeleton. The second subgroup contains two molecules, one being dehydroepiandrosterone (DHEA), with a modified unsaturation, and a 3-hydroxy function compared to the other 3-keto-4-androstene molecules of this group. This suggests that some modulating effects could be played by the position of the unsaturation. The third subgroup is more diverse since it includes estrone which has a small keto group at position C17 and an aromatic *A* ring. In this case, a modulating effect seems to be played by *A* ring aromaticity of the steroid molecule. It can be concluded that both the hindrance at the C17 position and the presence of unsaturated C-C bond within the *A* ring of steroid substrate play significant role for metabolism by CYP1A enzymes. The different features deduced from this analysis are summarized in Figure 4.

### 3.4. CYP1A Structural Elements Influencing Steroid Metabolism

**Figure 5:**
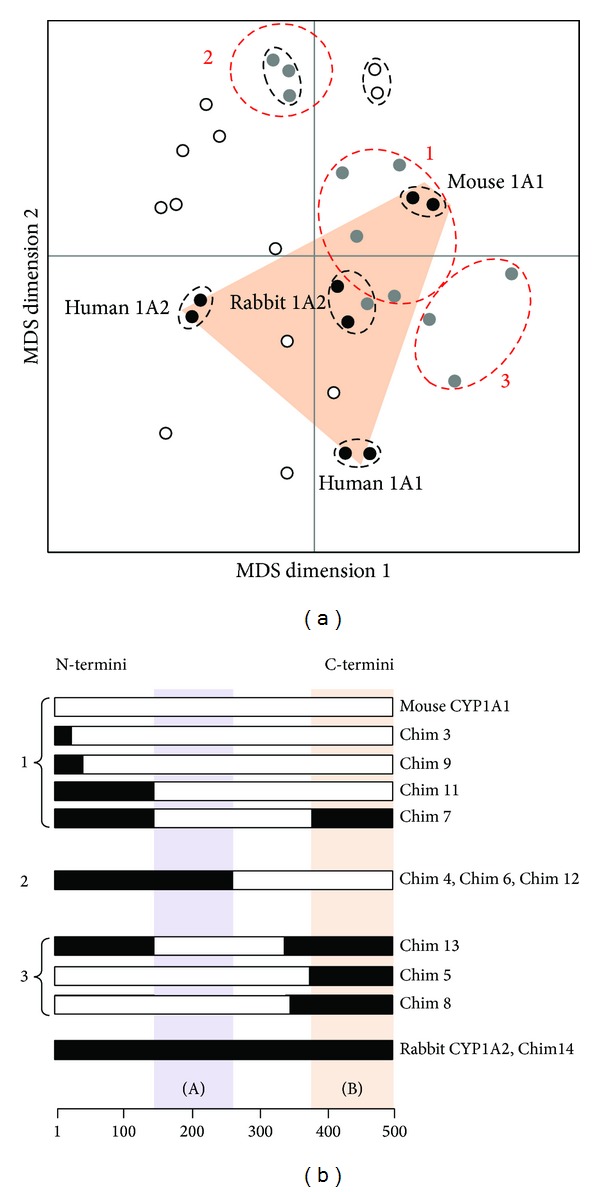
Panel (a), MDS configuration plot of the 31 CYP1A enzymes tested against all steroid activities. Some chimeric CYP1A enzymes are hidden replicates; that is, the cluster named “1” contains three chimeras of the Chim series which are triplicate of the same chimeric sequence. Solid circles correspond to wild-type enzymes, grey-filled circles to bi- or tripartite chimeras (Chim series), and open circles to mosaic chimeras (ChiMo series). The red ellipses correspond to cluster of enzymes which are discussed text based on their highly similar behaviour towards the steroids tested in this work. Panel (b), phylofunctional analysis of mouse-rabbit CYP1A chimeric enzymes assayed for hydroxylase activity with sixteen steroidal substrates. The open and solid bars represent, respectively, mouse CYP1A1 and rabbit CYP1A2 amino acid sequence segments. The purple and salmon boxes highlight the two elements of primary structure which are found to be correlated with the groups on the MDS plot.

When statistical analysis is no longer applied to the dataset columns (i.e., activities) but to rows (i.e., enzymes), it is possible to classify by MDS analysis the different wild-type and chimeric enzyme structures for their specificity toward the steroidal substrates assayed (Figure 5(a)). Human CYP1A2 enzyme exhibits a fairly narrow substrate specificity for steroids compared to the other tested wild-type CYP1A, even compared to its rabbit CYP1A2 ortholog. Consequently, the duplicated assays of this enzyme appear relatively isolated from its rabbit counterpart on the left border of the plot. Moreover, rabbit CYP1A2 presents a J helix which is of the 1A1-type in its sequence, contrary to what is seen for human CYP1A2 (see Table 1), and this helix has been proposed to be involved in the interaction with NADPH-P450 reductase [22, 23]. Notably, a systematic inversion of two charged residues is observed between CYP1A1s and CYP1A2s in this helix; namely, Arg 338 and Glu345 in human CYP1A1 correspond to Glu338 and Lys345 in human CYP1A2. In mouse CYP1A1, these two residues are conserved with human 1A1, namely, Arg342 and Glu349. But, when looking at rabbit CYP1A2 sequence, the situation observed is typical of CYP1A1 enzymes rather than CYP1A2 ones, with the combination of Arg338 and Glu345. This suggests that rabbit CYP1A2 is rather of the 1A1-type than of the 1A2-type and it could explain why rabbit CYP1A2 duplicate is found more closely aggregated with the two CYP1A1 clusters than with human CYP1A2 duplicate (Figure 5(a)).

Interestingly, the behaviors of chimeric enzymes do not appear as a linear combination of that of parental forms, a large part being outside of the boundary of polygon drawn from parental enzyme positions on the graph (Figure 5, salmon-colored area). Therefore, some novel steroid specificity is revealed within the collection of chimeric enzymes. Moreover, the chimeras escaping boundaries of wild-type specificities belong both to the Chim and the ChiMo series, indicating the absence of any clear correlation between the complexity of shuffling (number of crossovers) and the development of new activities. Independent replicas appear always tightly grouped on the graph, thus indicating that differences of behaviors between chimeras are not significantly influenced by experimental “noise.” A hidden triplicate of the same chimera (Chim4, Chim6, and Chim12) appears also as a tight aggregate on the MDS plot (red ellipse named “1”).

Several groups of enzymes can be drawn from this MDS plot. For the sake of clarity, this comparison was limited to wild-type enzymes and chimeric variants of the Chim series, since these variants exhibit a simpler shuffling in sequences than that of ChiMo series. On a MDS plot, if two objects (i.e., the enzymes in this work) are found close to each other, it means that their global behaviors toward the properties analyzed (i.e., the substrates in this work) are similar.

A first group, shown by a red ellipse denoted by “1” on the plot, contains wild-type mouse CYP1A1 and is located quite closely to the group of wild-type rabbit CYP1A2. One so-called “chimera,” Chim14, was found to be a hidden replicate of rabbit CYP1A2 and is thus aggregated to the rabbit CYP1A2 cluster. The two parental wild-type enzymes are found closely located. This indicates that their behaviors towards the steroidal substrates assayed in this work are quite similar.

Another, clearly outlying, group of chimeras is found in a quite remote area in the upper left part of the graph and is drawn in a red ellipse denoted by “2” in Figure 5(a). This group corresponds to a hidden triplicate of the same chimeric sequence and belongs to a larger group of more complex ChiMo chimeric structures whose representative points span the upper left quadrant. These sequences therefore encode CYP1A chimeric variants whose specificity profile for steroid substrates is very different from those of the parental wild-type enzymes.

The third group, found to be more slightly resolved from the group of wild-type enzymes, stands in the lower-right quadrant of the plot including three chimeras (Chim5, Chim8, and Chim13) of different sequences which is shown by the red ellipse denoted by “3.” Steroid specificity of group “3” enzymes moderately differs from the combination of the ones of parental enzymes (group “2”).

In a preliminary step to further analyze the relationships between global specificity towards steroids and sequence shuffling, Figure 5(b) illustrates a low resolution map of amino acid sequences of discussed chimera as compared to parental sequences. A simple segmentation identifies two sequence segments, designated as “A” and “B” (purple and salmon boxes on Figure 5(b)), whose sequence type varies accordingly to the global steroid specificity of the corresponding CYP1A enzyme. The sequence segment “A” encompasses amino acids ranging from residue 142 (mouse CYP1A1 numbering) to residue 247. The segment “B” encompasses amino acids from residue 377 to the C-terminus. It is apparent that the combination of the parental type of these two segments is determining of the functional groups, notably when segment “A” is of the 1A2-type and segment “B” of the 1A1-type. Moreover, the observation that chimeras in group 1 show a segment “B” either of the 1A1- or of the 1A2-type suggests that this segment is not critical for steroid metabolism but rather has modulating effect.

Altogether, sequence segments “A” and “B” represent almost 35% of the sequence, a rather large domain which needs further refinement to define the exact contributors. Segment “A” encompasses only one of the several SRSs, defined by Gotoh as P450 Substrate Recognition Sites [24], namely SRS2 which is highly divergent between 1A1 and 1A2 subtypes. SRS2 has also been highlighted frequently in controlling CYP1A enzymes functioning elsewhere [25–28]. Segment “B” encompasses two SRSs, namely, SRS5 and SRS6. Contrasting with segment “A,” these two SRSs are almost identical between mouse CYP1A1 and rabbit CYP1A2. Within SRS6, a threonine in mouse CYP1A1 is replaced by an isoleucine in rabbit CYP1A2 close to the C-terminus. This suggests that combinations of amino acids residues critically controlling steroid specificity in CYP1A enzymes should be more likely localized in segment “A” rather than in segment “B.”

**Figure 6:**
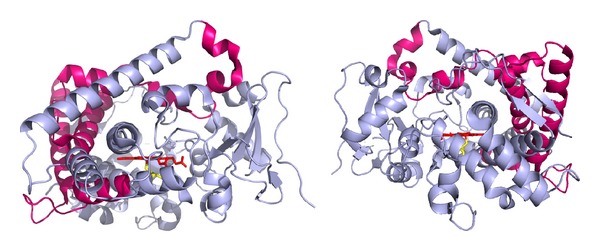
Human CYP1A1 shown in grey-blue ribbon structure (pdb entry 4i8v) highlighting sequence segment A (colored hot pink) and the heme (colored red). The two panels represent the structure rotated horizontally by 180° horizontally, with the I-helix viewed through its axis.

**Figure 7:**
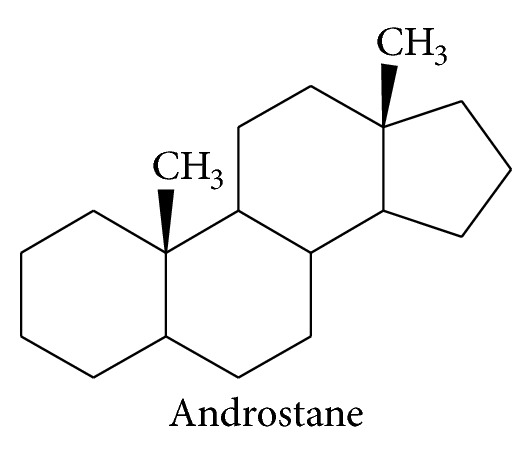


When placed on the crystal 3D structure of human CYP1A1, the “A” segment appears to be mostly located at the periphery of the protein molecule (Figure 6), whereas the helix I and its surrounding remain not affected by the shuffling taking place in Chim enzymes. It is consistent with the literature that helix I is crucial in P450 catalysis [11, 29] and that the distal cavity is not expected to be affected by sequence covering segment “A.” It is also consistent that sequence change in segment “B” could affect the lining of the proximal side of the heme and, thus, the heme-thiolate bond because it encompasses the cysteine that ligates the heme iron.

However, the way by which these structural factors can directly impact enzyme activity remains unclear based on structural data. Obviously, activity maps associated with all chimeras likely contain much more hidden information and the way to decipher this information in conjunction with structural data remains to be established on a more thorough theoretical ground. A potential approach would be solving experimentally the 3D structures of chimeric enzymes to understand how a novel activity pattern can be generated. This approach could be hampered by the fact that the comparison of the crystal structures of human CYP1A1 and human CYP1A2 parental enzymes was unfortunately poorly explanatory of functional differences since the two enzymes are highly similar in structure.

One interesting working hypothesis, supported by some preliminary data (not shown), would be that the apparent difference of specificity could more specifically result from differential control mechanisms of the substrate dependent initiation of oxygen activation cycle between CYP1A1s and CYP1A2s rather than from detailed features of substrate binding in the active site. In this hypothesis, the catalytic cycle of CYP1A1 would appear constitutively initiated even in the absence of substrate/ligand bound into the active site, whereas CYP1A2 would exhibit a more tight substrate dependent control of the initiation of the catalytic cycle. Such idea is suggested by preliminary data indicating that activity of chimeric structures supported by redox partners (NADPH-P450 reductase and cytochrome *b*_5_) or supported by hydroperoxide compounds, such as cumene hydroperoxide, can significantly differ for the same substrate depending on the presence of specific CYP1A2 sequence segments.

## 4. Conclusions

The approach initiated in this work takes profit of both a combinatorial mutagenesis to yield functional chimeric enzymes of increasing sequence shuffling complexity and of a combinatorial library of structurally related substrates having lateral groups of increasing complexity decorating a common simple chemical skeleton.

QSAR correlations may bring information on both the structural elements of the enzyme and the structural elements of the substrate that together govern recognition and regioselectivity in a crosstalk that remains to be understood. Several studies have illustrated the fact that CYP3A4 is one of the major players involved in the oxidation of steroid hormones in human liver microsomes [30], together with CYP2Cs [31]. Our work illustrated that members of the CYP1A family might play a complex role in hormonal regulation taking into account that CYP1A2 expression is mostly constitutive while that of CYP1A1 is highly inducible by a large range of xenobiotics, some of them being environmental pollutants and others being drugs [32].

Two important steroid structural features were characterized in this study, the substituent at the C17 position and the possibility for steroid molecules to bind in two opposite orientations within the catalytic site of CYP1A enzymes as evidenced by mifepristone activities. The nature and bulkiness of side groups at the C17 position clearly affect the catalytic efficiency of CYP1A enzymes.

Finally, the structural elements of the CYP1A protein that govern the parental phenotypes appear multiples, some combinations leading to the appearance of a novel substrate regiospecificity for steroids and related molecules. These determinants are mostly located at or near the protein surface and not close to the buried catalytic cavity suggesting some indirect mechanism controlling CYP1A activity. This result confirms previous works on P450 enzymes [33, 34] and opens new pathways for looking at sequence-activity relationships more deeply.

## Supplementary Material

Table S1. Table of specific activities observed with the different enzymes when assayed for the different steroidal substrates of this work (in five parts). EOR, MOR, EFEE stands for 7-ethoxy-, 7-methoxy-resorufin, and 7-ethoxy-fluorescein ethyl ester, respectively. The different hydroxylated metabolites observed in LC/MS are designated by one or two letters, abbreviatingthe name of the steroid, and a number corresponding to the rank in elution time on the LC. RUNdM stands for the unique N-demethylation metabolite of mifespristone observed in LC/MS. Specific activities are expressed as pmol product per min per mg microsomal protein for EOR, MOR and EFEE. Specific activities are expressed as LC/MS peak area units per min per mg micorosmal protein for all other activities.

## Figures and Tables

**Table 1:** Alignment of amino acid sequences of the J-helix of the four parental wild-type CYP1A enzymes studied in this work.

M m CYP1A1 341-PRVQRKIQEEL- 351	
Hs CYP1A1 337-...........- 347	
Hs CYP1A2 337- . EI.....K..- 347	
Oc CYP1A2 337-.RR.....E..- 347	
▲ ▲	

HS: *Homo sapiens*, Mm: *Mus musculus*, and Oc: *Oryctolagus cuniculus*. The solid triangles indicate the charged residue discussed in the text; a dot represents an amino acid residue identical to that of Mm CYP1A1 sequence.
