# Supplementary material for: High-Throughput Functional Screening of Steroid Substrates with Wild-Type and Chimeric P450 Enzymes
Source: Biomed Res Int. 2014 Aug 26;2014:764102. doi: 10.1155/2014/764102 (PMC4160651; doi:10.1155/2014/764102)
Supplement: Supplementary file 1 — Table S1. Table of specific activities observed with the different enzymes when assayed for the different steroidal substrates of this work (in five parts). EOR, MOR, EFEE stands for 7-ethoxy-, 7-methoxy-resorufin, and 7-ethoxy-fluorescein ethyl ester, respectively. The different hydroxylated metabolites observed in LC/MS are designated by one or two letters, abbreviatingthe name of the steroid, and a number corresponding to the rank in elution time on the LC. RUNdM stands for the unique N-demethylation metabolite of mifespristone observed in LC/MS. Specific activities are expressed as pmol product per min per mg microsomal protein for EOR, MOR and EFEE. Specific activities are expressed as LC/MS peak area units per min per mg micorosmal protein for all other activities. [file 764102.f1.pdf]

High-throughput functional screening of steroid substrates with wild-type and chimeric P450 enzymes

Philippe Urban, Gilles Truan, and Denis Pompon

# Supplementary Materials

**Table S1**. **Table of specific activities observed with the different enzymes when assayed for the different steroidal substrates of this work (in five parts)**. EOR, MOR, EFEE stands for 7-ethoxy-, 7-methoxy-resorufin, and 7-ethoxy-fluorescein ethyl ester, respectively. The different hydroxylated metabolites observed in LC/MS are designated by one or two letters, abbreviating the name of the steroid, and a number corresponding to the rank in elution time on the LC. RUNdM stands for the unique N-demethylation metabolite of mifespristone observed in LC/MS. Specific activities are expressed as pmol product per min per mg microsomal protein for EOR, MOR and EFEE. Specific activities are expressed as LC/MS peak area units per min per mg micorosmal protein for all other activities.

| **ENZYME** | | | | | TESTOSTERONE | | | | | MIFEPRISTONE | | | | | 17-METHYL-TESTOSTERONE | | | |
|---|---|---|---|---|---|---|---|---|---|---|---|---|---|---|---|---|---|---|
| | EOR | MOR | EFEE | T1 | T2 | T3 | T4 | RU NdM | RU1 | RU2 | RU3 | RU4 | RU5 | MT1 | MT2 | MT3 | MT4 | MT5 |
| Human wt CYP1A1-1 | 4,739E+03 | 1,352E+04 | 3,331E+02 | 1,721E+03 | 1,013E+02 | 0,000E+00 | 0,000E+00 | 8,361E+00 | 5,865E+02 | 1,216E+03 | 0,000E+00 | 1,215E+03 | 1,090E+03 | 1,291E+03 | 1,265E+02 | 7,483E+02 | 2,458E+03 | 0,000E+00 |
| Human wt CYP1A1-2 | 4,543E+03 | 1,361E+04 | 3,401E+02 | 1,436E+03 | 8,422E+01 | 0,000E+00 | 1,008E+03 | 1,355E+01 | 1,214E+03 | 8,811E+02 | 3,156E+04 | 2,292E+03 | 1,926E+03 | 2,159E+03 | 1,324E+02 | 4,353E+02 | 3,917E+03 | 0,000E+00 |
| Human wt CYP1A2-1 | 8,065E+02 | 1,230E+04 | 1,921E-01 | 0,000E+00 | 1,480E+00 | 0,000E+00 | 0,000E+00 | 4,531E-01 | 0,000E+00 | 0,000E+00 | 0,000E+00 | 0,000E+00 | 0,000E+00 | 0,000E+00 | 0,000E+00 | 0,000E+00 | 0,000E+00 | 0,000E+00 |
| Human wt CYP1A2-2 | 8,473E+02 | 1,311E+04 | 3,842E-01 | 0,000E+00 | 2,600E+00 | 0,000E+00 | 0,000E+00 | 7,681E-01 | 0,000E+00 | 0,000E+00 | 0,000E+00 | 0,000E+00 | 0,000E+00 | 0,000E+00 | 0,000E+00 | 0,000E+00 | 0,000E+00 | 0,000E+00 |
| Mouse wt CYP1A1-1 | 1,026E+04 | 3,567E+04 | 1,028E+02 | 1,713E+02 | 4,778E+01 | 0,000E+00 | 2,523E+03 | 4,961E+01 | 0,000E+00 | 0,000E+00 | 0,000E+00 | 0,000E+00 | 2,656E+02 | 0,000E+00 | 9,093E+01 | 6,635E+02 | 7,452E+02 | 0,000E+00 |
| Mouse wt CYP1A1-2 | 1,075E+04 | 3,888E+04 | 8,357E+01 | 9,606E+01 | 3,245E+01 | 0,000E+00 | 1,599E+03 | 6,963E+00 | 0,000E+00 | 0,000E+00 | 0,000E+00 | 0,000E+00 | 0,000E+00 | 0,000E+00 | 5,992E+01 | 4,167E+02 | 1,277E+03 | 0,000E+00 |
| Rabbit wt CYP1A2-1 | 2,408E+02 | 2,874E+04 | 3,202E+02 | 3,877E+02 | 5,880E+01 | 1,477E+02 | 0,000E+00 | 5,824E+01 | 0,000E+00 | 0,000E+00 | 0,000E+00 | 0,000E+00 | 0,000E+00 | 0,000E+00 | 7,068E+01 | 5,203E+02 | 4,353E+02 | 0,000E+00 |
| Rabbit wt CYP1A2-2 | 2,771E+02 | 2,767E+04 | 2,776E+02 | 9,673E+01 | 1,988E+01 | 1,664E+02 | 0,000E+00 | 1,463E+01 | 0,000E+00 | 0,000E+00 | 0,000E+00 | 0,000E+00 | 0,000E+00 | 0,000E+00 | 4,067E+01 | 4,488E+02 | 5,436E+02 | 0,000E+00 |
| Chim3 | 9,973E+03 | 3,055E+04 | 6,763E+01 | 0,000E+00 | 4,830E+01 | 3,442E+02 | 5,738E+03 | 1,057E+01 | 0,000E+00 | 0,000E+00 | 0,000E+00 | 0,000E+00 | 1,242E+02 | 0,000E+00 | 8,920E+01 | 6,494E+02 | 1,535E+03 | 0,000E+00 |
| Chim4 | 1,552E+03 | 1,315E+04 | 1,153E+02 | 6,767E+02 | 6,487E+01 | 3,155E+02 | 0,000E+00 | 9,386E+00 | 0,000E+00 | 0,000E+00 | 0,000E+00 | 0,000E+00 | 0,000E+00 | 0,000E+00 | 7,329E+01 | 2,625E+02 | 1,927E+03 | 0,000E+00 |
| Chim5 | 1,352E+04 | 3,210E+04 | 2,445E+02 | 1,177E+03 | 3,274E+02 | 0,000E+00 | 6,305E+03 | 4,961E+01 | 0,000E+00 | 0,000E+00 | 0,000E+00 | 5,631E+02 | 5,295E+02 | 0,000E+00 | 4,853E+02 | 4,282E+03 | 3,676E+02 | 0,000E+00 |
| Chim6 | 1,586E+03 | 1,295E+04 | 9,990E+01 | 6,932E+02 | 8,999E+01 | 3,792E+03 | 0,000E+00 | 8,518E+00 | 0,000E+00 | 0,000E+00 | 0,000E+00 | 0,000E+00 | 0,000E+00 | 0,000E+00 | 9,163E+01 | 4,851E+02 | 2,856E+03 | 0,000E+00 |
| Chim7 | 3,228E+03 | 1,622E+04 | 3,485E+02 | 0,000E+00 | 8,165E+01 | 0,000E+00 | 0,000E+00 | 9,632E+00 | 0,000E+00 | 0,000E+00 | 0,000E+00 | 0,000E+00 | 0,000E+00 | 0,000E+00 | 1,309E+02 | 1,814E+02 | 0,000E+00 | 0,000E+00 |
| Chim8 | 8,114E+03 | 1,450E+04 | 1,298E+02 | 0,000E+00 | 7,778E+01 | 0,000E+00 | 3,870E+03 | 3,220E+00 | 0,000E+00 | 0,000E+00 | 0,000E+00 | 0,000E+00 | 0,000E+00 | 0,000E+00 | 6,963E+01 | 8,440E+02 | 1,725E+03 | 0,000E+00 |
| Chim9 | 1,276E+04 | 4,494E+04 | 1,233E+02 | 0,000E+00 | 5,757E+01 | 0,000E+00 | 2,571E+03 | 1,943E+01 | 0,000E+00 | 0,000E+00 | 0,000E+00 | 0,000E+00 | 2,217E+02 | 0,000E+00 | 1,321E+02 | 1,201E+03 | 2,463E+03 | 0,000E+00 |
| Chim11 | 4,714E+03 | 2,401E+04 | 3,605E+02 | 1,629E+02 | 1,373E+01 | 0,000E+00 | 0,000E+00 | 5,190E+00 | 0,000E+00 | 0,000E+00 | 0,000E+00 | 0,000E+00 | 0,000E+00 | 0,000E+00 | 1,645E+01 | 0,000E+00 | 2,825E+02 | 0,000E+00 |
| Chim12 | 1,586E+03 | 1,295E+04 | 9,990E+01 | 6,932E+02 | 8,999E+01 | 4,131E+02 | 0,000E+00 | 8,518E+00 | 0,000E+00 | 0,000E+00 | 0,000E+00 | 0,000E+00 | 0,000E+00 | 0,000E+00 | 9,163E+01 | 4,851E+02 | 2,856E+03 | 0,000E+00 |
| Chim13 | 2,967E+03 | 9,834E+03 | 2,033E+02 | 0,000E+00 | 2,747E+01 | 7,882E+02 | 1,657E+03 | 2,837E+00 | 0,000E+00 | 0,000E+00 | 0,000E+00 | 0,000E+00 | 0,000E+00 | 0,000E+00 | 3,134E+01 | 6,238E+02 | 3,943E+02 | 0,000E+00 |
| Chim14 | 2,953E+02 | 3,439E+04 | 3,940E+02 | 0,000E+00 | 8,961E+00 | 0,000E+00 | 0,000E+00 | 8,564E+00 | 0,000E+00 | 0,000E+00 | 0,000E+00 | 0,000E+00 | 0,000E+00 | 0,000E+00 | 2,834E+01 | 3,284E+02 | 2,533E+02 | 0,000E+00 |
| ChiMo1 | 2,383E+03 | 1,188E+04 | 3,775E+01 | 1,966E+02 | 9,186E+00 | 0,000E+00 | 0,000E+00 | 9,133E+00 | 0,000E+00 | 0,000E+00 | 0,000E+00 | 0,000E+00 | 0,000E+00 | 0,000E+00 | 1,775E+01 | 0,000E+00 | 7,470E+02 | 0,000E+00 |
| ChiMo2 | 3,680E+02 | 1,461E+03 | 0,000E+00 | 0,000E+00 | 1,314E+00 | 0,000E+00 | 0,000E+00 | 0,000E+00 | 0,000E+00 | 0,000E+00 | 0,000E+00 | 0,000E+00 | 0,000E+00 | 0,000E+00 | 3,021E+00 | 0,000E+00 | 0,000E+00 | 0,000E+00 |
| ChiMo3 | 4,089E+01 | 5,233E+02 | 9,702E+00 | 0,000E+00 | 0,000E+00 | 0,000E+00 | 0,000E+00 | 2,218E-01 | 0,000E+00 | 0,000E+00 | 0,000E+00 | 0,000E+00 | 0,000E+00 | 0,000E+00 | 0,000E+00 | 0,000E+00 | 0,000E+00 | 0,000E+00 |
| ChiMo4 | 3,862E+01 | 2,835E+02 | 0,000E+00 | 0,000E+00 | 0,000E+00 | 0,000E+00 | 0,000E+00 | 0,000E+00 | 0,000E+00 | 0,000E+00 | 0,000E+00 | 0,000E+00 | 0,000E+00 | 0,000E+00 | 3,213E+00 | 0,000E+00 | 0,000E+00 | 0,000E+00 |
| ChiMo5 | 1,703E+04 | 5,059E+04 | 3,202E+02 | 2,581E+03 | 2,749E+02 | 2,462E+03 | 6,448E+03 | 1,563E+01 | 0,000E+00 | 0,000E+00 | 0,000E+00 | 4,633E+03 | 1,139E+03 | 9,220E+02 | 1,612E+02 | 1,618E+03 | 1,692E+03 | 0,000E+00 |
| ChiMo6-1 | 2,163E+03 | 1,167E+04 | 1,443E+02 | 0,000E+00 | 1,208E+01 | 0,000E+00 | 0,000E+00 | 9,175E-01 | 0,000E+00 | 0,000E+00 | 0,000E+00 | 0,000E+00 | 0,000E+00 | 0,000E+00 | 7,163E+00 | 0,000E+00 | 0,000E+00 | 3,087E+04 |
| ChiMo6-2 | 2,163E+03 | 1,167E+04 | 1,443E+02 | 1,396E+02 | 1,178E+01 | 1,705E+02 | 1,164E+03 | 0,000E+00 | 0,000E+00 | 0,000E+00 | 0,000E+00 | 0,000E+00 | 0,000E+00 | 0,000E+00 | 6,698E+00 | 0,000E+00 | 0,000E+00 | 5,306E+04 |
| ChiMo7 | 1,054E+04 | 5,526E+04 | 1,402E+02 | 0,000E+00 | 5,895E+01 | 2,138E+02 | 0,000E+00 | 3,661E+00 | 0,000E+00 | 0,000E+00 | 0,000E+00 | 0,000E+00 | 0,000E+00 | 0,000E+00 | 2,460E+01 | 8,690E+01 | 2,515E+02 | 0,000E+00 |
| ChiMo8 | 9,134E+03 | 3,399E+04 | 4,330E+02 | 1,455E+02 | 1,766E+02 | 9,471E+02 | 0,000E+00 | 5,355E+00 | 0,000E+00 | 0,000E+00 | 0,000E+00 | 0,000E+00 | 0,000E+00 | 0,000E+00 | 8,061E+01 | 2,853E+02 | 8,150E+02 | 0,000E+00 |
| ChiMo9 | 1,640E+03 | 9,943E+03 | 2,459E+01 | 1,227E+02 | 1,830E+01 | 8,479E+01 | 0,000E+00 | 5,128E+00 | 0,000E+00 | 0,000E+00 | 0,000E+00 | 0,000E+00 | 0,000E+00 | 0,000E+00 | 2,144E+01 | 2,975E+02 | 0,000E+00 | 0,000E+00 |
| ChiMo10 | 8,701E+02 | 5,102E+03 | 1,787E+01 | 0,000E+00 | 5,082E+00 | 0,000E+00 | 7,008E+03 | 0,000E+00 | 0,000E+00 | 0,000E+00 | 0,000E+00 | 0,000E+00 | 0,000E+00 | 0,000E+00 | 0,000E+00 | 0,000E+00 | 0,000E+00 | 0,000E+00 |
| ChiMo11 | 7,269E+01 | 1,047E+03 | 7,685E+00 | 0,000E+00 | 0,000E+00 | 0,000E+00 | 0,000E+00 | 0,000E+00 | 1,983E+03 | 1,173E+03 | 0,000E+00 | 0,000E+00 | 0,000E+00 | 0,000E+00 | 5,288E+00 | 0,000E+00 | 0,000E+00 | 0,000E+00 |

| ENZYME | | | CORTEXOLONE | | | | | PROGESTERONE | | | | | | CORTICOSTERONE | | |
|---|---|---|---|---|---|---|---|---|---|---|---|---|---|---|---|---|
| | CTX1 | CTX2 | CTX3 | CTX4 | CTX5 | PROG1 | PROG2 | PROG3 | PROG4 | PROG5 | PROG6 | CTC1 | CTC2 | CTC3 | CTC4 | CTC5 |
| Human wt CYP1A1-1 | 2,641E+02 | 0,000E+00 | 0,000E+00 | 0,000E+00 | 1,387E+02 | 1,014E+02 | 0,000E+00 | 3,132E+01 | 2,298E+02 | 2,184E+02 | 0,000E+00 | 1,856E+02 | 0,000E+00 | 0,000E+00 | 0,000E+00 | 2,859E+02 |
| Human wt CYP1A1-2 | 2,956E+02 | 0,000E+00 | 0,000E+00 | 0,000E+00 | 1,873E+02 | 7,828E+01 | 0,000E+00 | 1,793E+01 | 1,292E+02 | 1,681E+02 | 6,222E+02 | 1,696E+02 | 0,000E+00 | 0,000E+00 | 0,000E+00 | 1,796E+02 |
| Human wt CYP1A2-1 | 0,000E+00 | 0,000E+00 | 0,000E+00 | 0,000E+00 | 3,250E+01 | 0,000E+00 | 0,000E+00 | 0,000E+00 | 1,249E+01 | 4,243E+01 | 8,314E+02 | 0,000E+00 | 0,000E+00 | 0,000E+00 | 0,000E+00 | 0,000E+00 |
| Human wt CYP1A2-2 | 0,000E+00 | 0,000E+00 | 0,000E+00 | 0,000E+00 | 2,490E+01 | 0,000E+00 | 0,000E+00 | 1,662E-01 | 1,417E+01 | 2,813E+01 | 6,204E+02 | 0,000E+00 | 0,000E+00 | 0,000E+00 | 0,000E+00 | 0,000E+00 |
| Mouse wt CYP1A1-1 | 5,731E+02 | 0,000E+00 | 0,000E+00 | 0,000E+00 | 1,266E+02 | 1,165E+01 | 0,000E+00 | 1,765E+01 | 1,619E+02 | 1,221E+02 | 5,500E+02 | 1,586E+02 | 1,302E+03 | 2,155E+03 | 0,000E+00 | 8,990E+01 |
| Mouse wt CYP1A1-2 | 2,850E+02 | 0,000E+00 | 0,000E+00 | 0,000E+00 | 3,920E+01 | 4,349E+00 | 0,000E+00 | 9,353E+00 | 3,296E+01 | 1,114E+01 | 5,864E+02 | 7,690E+01 | 0,000E+00 | 9,091E+02 | 0,000E+00 | 5,966E+01 |
| Rabbit wt CYP1A2-1 | 4,777E+02 | 6,227E+03 | 0,000E+00 | 0,000E+00 | 1,689E+02 | 1,707E+01 | 0,000E+00 | 2,524E+01 | 3,365E+02 | 4,870E+02 | 7,394E+02 | 2,151E+02 | 0,000E+00 | 3,345E+03 | 5,812E+04 | 6,061E+01 |
| Rabbit wt CYP1A2-2 | 1,472E+02 | 0,000E+00 | 0,000E+00 | 0,000E+00 | 5,377E+01 | 4,093E+00 | 0,000E+00 | 1,173E+01 | 1,160E+02 | 1,782E+02 | 9,434E+01 | 4,414E+01 | 0,000E+00 | 1,593E+03 | 0,000E+00 | 2,836E+01 |
| Chim3 | 0,000E+00 | 7,763E+03 | 0,000E+00 | 1,862E+04 | 1,023E+02 | 1,205E+01 | 0,000E+00 | 1,906E+01 | 1,345E+02 | 5,319E+01 | 1,021E+03 | 3,244E+02 | 0,000E+00 | 0,000E+00 | 0,000E+00 | 8,795E+01 |
| Chim4 | 1,353E+02 | 0,000E+00 | 0,000E+00 | 0,000E+00 | 4,066E+02 | 3,278E+01 | 0,000E+00 | 1,948E+01 | 5,681E+02 | 1,084E+02 | 1,867E+03 | 7,250E+01 | 0,000E+00 | 0,000E+00 | 0,000E+00 | 6,462E+01 |
| Chim5 | 2,737E+03 | 3,458E+04 | 5,601E+04 | 2,999E+04 | 7,752E+02 | 8,165E+01 | 0,000E+00 | 1,810E+02 | 9,229E+02 | 5,503E+02 | 0,000E+00 | 1,577E+03 | 1,427E+04 | 1,596E+04 | 0,000E+00 | 7,440E+02 |
| Chim6 | 3,546E+02 | 0,000E+00 | 0,000E+00 | 0,000E+00 | 4,494E+02 | 4,020E+01 | 0,000E+00 | 3,432E+01 | 6,463E+02 | 1,731E+02 | 2,188E+03 | 2,000E+02 | 0,000E+00 | 0,000E+00 | 0,000E+00 | 9,900E+01 |
| Chim7 | 5,563E+02 | 0,000E+00 | 0,000E+00 | 0,000E+00 | 2,628E+02 | 5,783E+01 | 0,000E+00 | 2,511E+01 | 1,773E+02 | 8,876E+01 | 0,000E+00 | 5,340E+01 | 1,851E+03 | 1,262E+03 | 0,000E+00 | 1,118E+02 |
| Chim8 | 3,504E+02 | 0,000E+00 | 0,000E+00 | 0,000E+00 | 9,859E+01 | 6,602E+00 | 0,000E+00 | 1,862E+01 | 0,000E+00 | 1,025E+02 | 0,000E+00 | 5,050E+01 | 0,000E+00 | 0,000E+00 | 0,000E+00 | 4,671E+01 |
| Chim9 | 3,114E+02 | 0,000E+00 | 0,000E+00 | 0,000E+00 | 1,660E+02 | 1,373E+01 | 0,000E+00 | 2,614E+01 | 3,320E+02 | 0,000E+00 | 1,468E+03 | 2,222E+02 | 0,000E+00 | 2,093E+03 | 0,000E+00 | 1,370E+02 |
| Chim11 | 2,409E+02 | 0,000E+00 | 0,000E+00 | 0,000E+00 | 9,163E+01 | 8,500E+00 | 0,000E+00 | 1,688E+00 | 1,004E+01 | 0,000E+00 | 5,126E+02 | 0,000E+00 | 0,000E+00 | 0,000E+00 | 0,000E+00 | 2,491E+01 |
| Chim12 | 3,546E+02 | 0,000E+00 | 0,000E+00 | 0,000E+00 | 4,494E+02 | 4,020E+01 | 0,000E+00 | 3,432E+01 | 6,623E+02 | 1,731E+02 | 2,188E+03 | 2,000E+02 | 0,000E+00 | 0,000E+00 | 0,000E+00 | 9,900E+01 |
| Chim13 | 1,781E+02 | 0,000E+00 | 0,000E+00 | 0,000E+00 | 6,798E+01 | 0,000E+00 | 0,000E+00 | 4,589E+00 | 9,957E+01 | 9,236E+02 | 0,000E+00 | 2,029E+01 | 0,000E+00 | 0,000E+00 | 0,000E+00 | 9,206E+01 |
| Chim14 | 1,090E+02 | 0,000E+00 | 0,000E+00 | 0,000E+00 | 7,060E+01 | 6,051E+00 | 0,000E+00 | 6,980E+00 | 8,318E+01 | 1,029E+02 | 0,000E+00 | 9,457E+00 | 0,000E+00 | 0,000E+00 | 0,000E+00 | 6,007E+00 |
| ChiMo1 | 0,000E+00 | 0,000E+00 | 0,000E+00 | 0,000E+00 | 8,749E+01 | 1,225E+01 | 0,000E+00 | 7,300E+00 | 5,555E+01 | 2,043E+02 | 1,468E+03 | 7,837E+01 | 0,000E+00 | 0,000E+00 | 0,000E+00 | 2,584E+01 |
| ChiMo2 | 0,000E+00 | 0,000E+00 | 0,000E+00 | 0,000E+00 | 1,548E+02 | 9,077E-01 | 0,000E+00 | 4,145E-01 | 0,000E+00 | 1,372E+02 | 3,414E+02 | 0,000E+00 | 0,000E+00 | 0,000E+00 | 0,000E+00 | 3,621E+00 |
| ChiMo3 | 9,204E+01 | 0,000E+00 | 0,000E+00 | 0,000E+00 | 4,380E+01 | 0,000E+00 | 0,000E+00 | 0,000E+00 | 0,000E+00 | 0,000E+00 | 0,000E+00 | 7,845E+00 | 0,000E+00 | 0,000E+00 | 0,000E+00 | 1,726E+01 |
| ChiMo4 | 0,000E+00 | 0,000E+00 | 0,000E+00 | 0,000E+00 | 2,132E+01 | 0,000E+00 | 0,000E+00 | 0,000E+00 | 0,000E+00 | 0,000E+00 | 0,000E+00 | 0,000E+00 | 0,000E+00 | 0,000E+00 | 0,000E+00 | 1,450E+01 |
| ChiMo5 | 0,000E+00 | 0,000E+00 | 0,000E+00 | 0,000E+00 | 2,864E+02 | 1,496E+02 | 0,000E+00 | 2,881E+01 | 2,604E+02 | 1,880E+02 | 0,000E+00 | 6,558E+01 | 0,000E+00 | 0,000E+00 | 0,000E+00 | 2,131E+02 |
| ChiMo6-1 | 2,252E+02 | 0,000E+00 | 0,000E+00 | 0,000E+00 | 1,053E+02 | 3,605E+01 | 0,000E+00 | 2,251E+00 | 2,504E+01 | 1,466E+01 | 5,173E+02 | 1,790E+01 | 0,000E+00 | 0,000E+00 | 0,000E+00 | 1,090E+02 |
| ChiMo6-2 | 0,000E+00 | 0,000E+00 | 0,000E+00 | 0,000E+00 | 1,020E+02 | 2,223E+01 | 0,000E+00 | 6,074E-01 | 0,000E+00 | 0,000E+00 | 1,243E+03 | 0,000E+00 | 0,000E+00 | 0,000E+00 | 0,000E+00 | 2,667E+01 |
| ChiMo7 | 0,000E+00 | 0,000E+00 | 0,000E+00 | 0,000E+00 | 3,731E+02 | 1,620E+02 | 0,000E+00 | 7,669E+00 | 1,167E+02 | 7,321E+01 | 6,829E+02 | 0,000E+00 | 0,000E+00 | 0,000E+00 | 0,000E+00 | 3,176E+02 |
| ChiMo8 | 2,735E+02 | 0,000E+00 | 0,000E+00 | 3,120E+04 | 4,373E+02 | 9,868E+01 | 8,557E+03 | 3,812E+01 | 4,095E+02 | 7,670E+02 | 8,919E+02 | 1,169E+02 | 0,000E+00 | 1,531E+03 | 0,000E+00 | 2,810E+02 |
| ChiMo9 | 1,374E+02 | 0,000E+00 | 0,000E+00 | 0,000E+00 | 1,312E+02 | 5,751E+00 | 2,284E+03 | 4,929E+00 | 4,898E+01 | 0,000E+00 | 1,029E+03 | 7,126E+01 | 0,000E+00 | 0,000E+00 | 0,000E+00 | 9,490E+01 |
| ChiMo10 | 0,000E+00 | 0,000E+00 | 0,000E+00 | 0,000E+00 | 5,715E+01 | 2,239E+01 | 0,000E+00 | 1,084E+01 | 1,010E+02 | 1,322E+03 | 0,000E+00 | 1,111E+01 | 0,000E+00 | 0,000E+00 | 0,000E+00 | 1,592E+01 |
| ChiMo11 | 0,000E+00 | 0,000E+00 | 0,000E+00 | 0,000E+00 | 0,000E+00 | 0,000E+00 | 0,000E+00 | 0,000E+00 | 0,000E+00 | 0,000E+00 | 0,000E+00 | 0,000E+00 | 0,000E+00 | 0,000E+00 | 0,000E+00 | 0,000E+00 |

| **ENZYME** | | | PREGNENOLONE | | | | | | 17-HYDROXY-PROGESTERONE | | | | | DHEA | | |
|---|---|---|---|---|---|---|---|---|---|---|---|---|---|---|---|---|
| | PREG1 | PREG2 | PREG3 | PREG4 | PREG5 | PREG6 | PREG7 | 17HP1 | 17HP2 | 17HP3 | 17HP4 | 17HP5 | DHEA1 | DHEA2 | DHEA3 | DHEA4 |
| Human wt CYP1A1-1 | 8,557E+02 | 0,000E+00 | 3,414E+03 | 0,000E+00 | 0,000E+00 | 1,571E+03 | 2,624E+02 | 3,943E+02 | 0,000E+00 | 0,000E+00 | 8,514E+02 | 0,000E+00 | 1,154E+03 | 1,124E+03 | 0,000E+00 | 2,113E+02 |
| Human wt CYP1A1-2 | 3,788E+02 | 0,000E+00 | 1,432E+03 | 0,000E+00 | 0,000E+00 | 8,867E+02 | 3,204E+02 | 1,377E+02 | 0,000E+00 | 0,000E+00 | 9,551E+02 | 0,000E+00 | 4,947E+02 | 1,075E+03 | 0,000E+00 | 2,264E+02 |
| Human wt CYP1A2-1 | 0,000E+00 | 0,000E+00 | 0,000E+00 | 0,000E+00 | 0,000E+00 | 0,000E+00 | 0,000E+00 | 0,000E+00 | 0,000E+00 | 0,000E+00 | 0,000E+00 | 0,000E+00 | 0,000E+00 | 0,000E+00 | 0,000E+00 | 5,851E+02 |
| Human wt CYP1A2-2 | 0,000E+00 | 0,000E+00 | 0,000E+00 | 0,000E+00 | 0,000E+00 | 0,000E+00 | 0,000E+00 | 0,000E+00 | 0,000E+00 | 0,000E+00 | 0,000E+00 | 0,000E+00 | 0,000E+00 | 0,000E+00 | 0,000E+00 | 1,737E+02 |
| Mouse wt CYP1A1-1 | 5,354E+01 | 0,000E+00 | 0,000E+00 | 0,000E+00 | 0,000E+00 | 2,270E+02 | 0,000E+00 | 1,218E+02 | 0,000E+00 | 0,000E+00 | 0,000E+00 | 0,000E+00 | 6,415E+02 | 0,000E+00 | 0,000E+00 | 0,000E+00 |
| Mouse wt CYP1A1-2 | 4,742E+01 | 0,000E+00 | 0,000E+00 | 0,000E+00 | 2,811E+02 | 2,734E+02 | 0,000E+00 | 1,560E+02 | 0,000E+00 | 0,000E+00 | 0,000E+00 | 0,000E+00 | 2,580E+02 | 2,356E+02 | 0,000E+00 | 0,000E+00 |
| Rabbit wt CYP1A2-1 | 3,107E+01 | 0,000E+00 | 0,000E+00 | 0,000E+00 | 0,000E+00 | 0,000E+00 | 2,367E+02 | 1,957E+02 | 0,000E+00 | 0,000E+00 | 0,000E+00 | 0,000E+00 | 6,043E+02 | 4,626E+02 | 0,000E+00 | 4,624E+02 |
| Rabbit wt CYP1A2-2 | 0,000E+00 | 0,000E+00 | 0,000E+00 | 0,000E+00 | 0,000E+00 | 0,000E+00 | 5,756E+01 | 1,815E+01 | 0,000E+00 | 0,000E+00 | 0,000E+00 | 0,000E+00 | 9,974E+01 | 1,236E+02 | 0,000E+00 | 1,537E+02 |
| Chim3 | 9,131E+01 | 0,000E+00 | 2,945E+02 | 0,000E+00 | 0,000E+00 | 0,000E+00 | 0,000E+00 | 1,866E+02 | 0,000E+00 | 0,000E+00 | 0,000E+00 | 0,000E+00 | 8,577E+02 | 9,453E+02 | 0,000E+00 | 0,000E+00 |
| Chim4 | 2,890E+01 | 2,734E+03 | 3,951E+02 | 0,000E+00 | 0,000E+00 | 3,770E+02 | 7,834E+02 | 1,769E+02 | 0,000E+00 | 0,000E+00 | 2,720E+02 | 0,000E+00 | 1,165E+03 | 1,215E+03 | 0,000E+00 | 2,073E+02 |
| Chim5 | 2,429E+02 | 0,000E+00 | 3,299E+03 | 0,000E+00 | 7,415E+03 | 3,252E+03 | 1,564E+02 | 1,669E+03 | 1,364E+04 | 2,435E+04 | 7,770E+02 | 0,000E+00 | 2,003E+03 | 2,000E+03 | 0,000E+00 | 3,847E+02 |
| Chim6 | 6,768E+01 | 1,378E+04 | 0,000E+00 | 0,000E+00 | 0,000E+00 | 4,927E+02 | 9,095E+02 | 3,623E+02 | 0,000E+00 | 0,000E+00 | 3,416E+02 | 0,000E+00 | 3,006E+03 | 2,070E+03 | 0,000E+00 | 5,747E+02 |
| Chim7 | 1,610E+02 | 0,000E+00 | 3,331E+02 | 0,000E+00 | 0,000E+00 | 7,085E+02 | 0,000E+00 | 5,509E+02 | 0,000E+00 | 0,000E+00 | 2,955E+02 | 0,000E+00 | 4,237E+02 | 5,731E+02 | 0,000E+00 | 0,000E+00 |
| Chim8 | 0,000E+00 | 0,000E+00 | 0,000E+00 | 0,000E+00 | 0,000E+00 | 1,516E+02 | 0,000E+00 | 8,449E+01 | 0,000E+00 | 0,000E+00 | 0,000E+00 | 0,000E+00 | 0,000E+00 | 0,000E+00 | 0,000E+00 | 0,000E+00 |
| Chim9 | 7,503E+01 | 8,500E+03 | 7,526E+02 | 0,000E+00 | 0,000E+00 | 2,578E+02 | 0,000E+00 | 3,974E+02 | 0,000E+00 | 0,000E+00 | 1,089E+02 | 0,000E+00 | 1,187E+03 | 7,101E+02 | 0,000E+00 | 1,348E+02 |
| Chim11 | 1,437E+01 | 0,000E+00 | 0,000E+00 | 0,000E+00 | 0,000E+00 | 0,000E+00 | 0,000E+00 | 1,428E+02 | 0,000E+00 | 0,000E+00 | 2,200E+02 | 0,000E+00 | 3,063E+02 | 1,151E+02 | 0,000E+00 | 0,000E+00 |
| Chim12 | 6,768E+01 | 1,378E+04 | 0,000E+00 | 0,000E+00 | 0,000E+00 | 4,927E+02 | 9,095E+02 | 3,623E+02 | 0,000E+00 | 0,000E+00 | 3,416E+02 | 0,000E+00 | 3,006E+03 | 2,070E+03 | 0,000E+00 | 5,747E+02 |
| Chim13 | 1,751E+01 | 0,000E+00 | 0,000E+00 | 0,000E+00 | 0,000E+00 | 6,961E+01 | 0,000E+00 | 8,492E+01 | 0,000E+00 | 0,000E+00 | 0,000E+00 | 0,000E+00 | 0,000E+00 | 0,000E+00 | 0,000E+00 | 0,000E+00 |
| Chim14 | 0,000E+00 | 0,000E+00 | 0,000E+00 | 0,000E+00 | 0,000E+00 | 0,000E+00 | 0,000E+00 | 5,028E+01 | 0,000E+00 | 0,000E+00 | 0,000E+00 | 0,000E+00 | 0,000E+00 | 0,000E+00 | 0,000E+00 | 0,000E+00 |
| ChiMo1 | 0,000E+00 | 0,000E+00 | 0,000E+00 | 0,000E+00 | 0,000E+00 | 9,374E+01 | 5,035E+01 | 0,000E+00 | 0,000E+00 | 0,000E+00 | 0,000E+00 | 2,759E+05 | 0,000E+00 | 0,000E+00 | 0,000E+00 | 1,950E+02 |
| ChiMo2 | 0,000E+00 | 0,000E+00 | 0,000E+00 | 0,000E+00 | 0,000E+00 | 0,000E+00 | 0,000E+00 | 0,000E+00 | 0,000E+00 | 0,000E+00 | 0,000E+00 | 0,000E+00 | 0,000E+00 | 0,000E+00 | 0,000E+00 | 0,000E+00 |
| ChiMo3 | 0,000E+00 | 0,000E+00 | 0,000E+00 | 0,000E+00 | 0,000E+00 | 0,000E+00 | 0,000E+00 | 0,000E+00 | 0,000E+00 | 0,000E+00 | 0,000E+00 | 0,000E+00 | 0,000E+00 | 0,000E+00 | 0,000E+00 | 0,000E+00 |
| ChiMo4 | 0,000E+00 | 0,000E+00 | 0,000E+00 | 0,000E+00 | 0,000E+00 | 0,000E+00 | 0,000E+00 | 0,000E+00 | 0,000E+00 | 0,000E+00 | 0,000E+00 | 0,000E+00 | 0,000E+00 | 0,000E+00 | 0,000E+00 | 0,000E+00 |
| ChiMo5 | 1,096E+03 | 0,000E+00 | 3,093E+03 | 1,188E+05 | 2,962E+03 | 2,026E+03 | 5,249E+02 | 2,807E+02 | 0,000E+00 | 0,000E+00 | 2,568E+03 | 0,000E+00 | 2,733E+03 | 2,123E+03 | 0,000E+00 | 5,031E+02 |
| ChiMo6-1 | 3,392E+01 | 0,000E+00 | 0,000E+00 | 0,000E+00 | 0,000E+00 | 0,000E+00 | 3,755E+01 | 2,746E+01 | 0,000E+00 | 0,000E+00 | 5,299E+02 | 0,000E+00 | 0,000E+00 | 0,000E+00 | 0,000E+00 | 0,000E+00 |
| ChiMo6-2 | 0,000E+00 | 0,000E+00 | 0,000E+00 | 0,000E+00 | 0,000E+00 | 0,000E+00 | 0,000E+00 | 0,000E+00 | 0,000E+00 | 0,000E+00 | 3,080E+02 | 0,000E+00 | 0,000E+00 | 0,000E+00 | 0,000E+00 | 0,000E+00 |
| ChiMo7 | 2,086E+02 | 0,000E+00 | 1,217E+03 | 0,000E+00 | 0,000E+00 | 7,121E+02 | 1,346E+02 | 0,000E+00 | 0,000E+00 | 0,000E+00 | 2,434E+03 | 0,000E+00 | 0,000E+00 | 0,000E+00 | 0,000E+00 | 1,052E+02 |
| ChiMo8 | 5,190E+02 | 0,000E+00 | 3,840E+03 | 0,000E+00 | 2,230E+03 | 1,745E+03 | 5,125E+02 | 1,339E+03 | 8,330E+03 | 1,093E+04 | 4,544E+03 | 0,000E+00 | 1,751E+02 | 6,657E+02 | 2,964E+03 | 7,209E+02 |
| ChiMo9 | 3,652E+01 | 0,000E+00 | 3,069E+02 | 0,000E+00 | 0,000E+00 | 0,000E+00 | 5,468E+01 | 0,000E+00 | 0,000E+00 | 0,000E+00 | 0,000E+00 | 0,000E+00 | 0,000E+00 | 0,000E+00 | 0,000E+00 | 0,000E+00 |
| ChiMo10 | 0,000E+00 | 0,000E+00 | 1,223E+02 | 0,000E+00 | 2,989E+02 | 9,269E+01 | 0,000E+00 | 0,000E+00 | 0,000E+00 | 0,000E+00 | 0,000E+00 | 0,000E+00 | 0,000E+00 | 1,089E+02 | 0,000E+00 | 2,824E+03 |
| ChiMo11 | 0,000E+00 | 0,000E+00 | 0,000E+00 | 0,000E+00 | 0,000E+00 | 0,000E+00 | 0,000E+00 | 0,000E+00 | 0,000E+00 | 0,000E+00 | 0,000E+00 | 0,000E+00 | 0,000E+00 | 0,000E+00 | 0,000E+00 | 0,000E+00 |

| ENZYME | | 21-HYDROXY-PROGESTERONE | | | | | ESTRONE | | | | | | NOOTKATONE | | | | |
|---|---|---|---|---|---|---|---|---|---|---|---|---|---|---|---|---|---|
| | 21HP1 | 21HP2 | 21HP3 | 21HP4 | 21HP5 | 21HP6 | EST1 | EST2 | EST3 | EST4 | NTK1 | NTK2 | NTK3 | NTK4 | NTK5 | NTK6 | NTK7 | NTK8 |
| Human wt CYP1A1-1 | 8,201E+01 | 0,000E+00 | 1,027E+04 | 1,590E+02 | 0,000E+00 | 0,000E+00 | 3,557E+02 | 0,000E+00 | 0,000E+00 | 0,000E+00 | 1,703E+03 | 5,346E+02 | 6,646E+02 | 0,000E+00 | 5,317E+02 | 1,043E+02 | 1,510E+03 | 0,000E+00 |
| Human wt CYP1A1-2 | 3,342E+01 | 0,000E+00 | 4,049E+03 | 1,600E+02 | 0,000E+00 | 0,000E+00 | 2,656E+02 | 0,000E+00 | 0,000E+00 | 0,000E+00 | 1,815E+03 | 4,008E+03 | 4,317E+02 | 2,657E+03 | 4,774E+02 | 5,539E+01 | 7,128E+02 | 0,000E+00 |
| Human wt CYP1A2-1 | 0,000E+00 | 0,000E+00 | 0,000E+00 | 6,175E+00 | 0,000E+00 | 0,000E+00 | 0,000E+00 | 0,000E+00 | 0,000E+00 | 0,000E+00 | 0,000E+00 | 0,000E+00 | 0,000E+00 | 0,000E+00 | 0,000E+00 | 0,000E+00 | 0,000E+00 | 1,139E+03 |
| Human wt CYP1A2-2 | 0,000E+00 | 0,000E+00 | 0,000E+00 | 1,216E+01 | 0,000E+00 | 0,000E+00 | 0,000E+00 | 0,000E+00 | 0,000E+00 | 0,000E+00 | 0,000E+00 | 0,000E+00 | 0,000E+00 | 0,000E+00 | 0,000E+00 | 0,000E+00 | 0,000E+00 | 2,337E+02 |
| Mouse wt CYP1A1-1 | 4,745E+01 | 0,000E+00 | 0,000E+00 | 2,327E+01 | 1,275E+02 | 0,000E+00 | 1,626E+02 | 3,351E+03 | 0,000E+00 | 0,000E+00 | 6,545E+02 | 0,000E+00 | 1,879E+02 | 0,000E+00 | 1,439E+02 | 6,163E+01 | 8,522E+02 | 3,051E+02 |
| Mouse wt CYP1A1-2 | 1,541E+01 | 0,000E+00 | 0,000E+00 | 1,547E+01 | 0,000E+00 | 1,512E+03 | 7,765E+01 | 3,406E+03 | 0,000E+00 | 0,000E+00 | 0,000E+00 | 0,000E+00 | 1,162E+02 | 0,000E+00 | 1,518E+02 | 3,087E+01 | 2,258E+02 | 9,098E+01 |
| Rabbit wt CYP1A2-1 | 1,187E+02 | 0,000E+00 | 0,000E+00 | 4,669E+01 | 5,247E+02 | 2,420E+03 | 1,326E+01 | 0,000E+00 | 0,000E+00 | 0,000E+00 | 0,000E+00 | 0,000E+00 | 5,313E+02 | 0,000E+00 | 0,000E+00 | 3,410E+01 | 4,006E+02 | 1,864E+03 |
| Rabbit wt CYP1A2-2 | 5,218E+01 | 0,000E+00 | 0,000E+00 | 1,848E+01 | 1,323E+02 | 4,213E+02 | 5,413E+00 | 0,000E+00 | 0,000E+00 | 0,000E+00 | 0,000E+00 | 0,000E+00 | 1,706E+02 | 0,000E+00 | 8,883E+01 | 1,086E+01 | 3,233E+02 | 3,833E+02 |
| Chim3 | 4,460E+01 | 0,000E+00 | 0,000E+00 | 2,348E+01 | 4,469E+02 | 0,000E+00 | 1,838E+02 | 2,856E+03 | 0,000E+00 | 4,630E+04 | 0,000E+00 | 0,000E+00 | 1,486E+02 | 0,000E+00 | 1,497E+02 | 5,648E+01 | 7,771E+02 | 2,584E+01 |
| Chim4 | 7,288E+01 | 0,000E+00 | 2,232E+03 | 7,134E+01 | 0,000E+00 | 0,000E+00 | 7,084E+00 | 0,000E+00 | 0,000E+00 | 0,000E+00 | 6,245E+02 | 3,399E+02 | 1,026E+02 | 0,000E+00 | 4,553E+01 | 5,671E+01 | 2,293E+02 | 0,000E+00 |
| Chim5 | 6,402E+02 | 1,176E+04 | 0,000E+00 | 2,571E+02 | 0,000E+00 | 0,000E+00 | 2,871E+02 | 8,003E+03 | 0,000E+00 | 0,000E+00 | 3,698E+03 | 1,308E+03 | 1,510E+03 | 0,000E+00 | 9,339E+02 | 2,861E+02 | 2,129E+03 | 1,456E+03 |
| Chim6 | 1,480E+02 | 3,180E+03 | 0,000E+00 | 1,065E+02 | 0,000E+00 | 0,000E+00 | 1,171E+01 | 0,000E+00 | 0,000E+00 | 0,000E+00 | 4,658E+02 | 0,000E+00 | 2,553E+02 | 0,000E+00 | 9,402E+01 | 7,452E+01 | 2,874E+02 | 1,521E+02 |
| Chim7 | 4,217E+01 | 0,000E+00 | 0,000E+00 | 6,228E+01 | 5,896E+02 | 1,563E+03 | 3,212E+01 | 3,380E+02 | 0,000E+00 | 0,000E+00 | 0,000E+00 | 0,000E+00 | 2,063E+02 | 0,000E+00 | 2,362E+02 | 3,632E+01 | 0,000E+00 | 0,000E+00 |
| Chim8 | 8,109E+01 | 0,000E+00 | 0,000E+00 | 2,147E+01 | 0,000E+00 | 1,858E+03 | 4,969E+01 | 0,000E+00 | 0,000E+00 | 0,000E+00 | 4,947E+02 | 0,000E+00 | 1,070E+02 | 0,000E+00 | 1,509E+03 | 5,174E+01 | 0,000E+00 | 0,000E+00 |
| Chim9 | 8,228E+01 | 0,000E+00 | 0,000E+00 | 3,626E+01 | 4,555E+02 | 2,116E+03 | 2,257E+02 | 4,704E+03 | 0,000E+00 | 0,000E+00 | 0,000E+00 | 0,000E+00 | 1,583E+02 | 0,000E+00 | 1,078E+02 | 1,036E+02 | 7,369E+02 | 0,000E+00 |
| Chim11 | 4,605E+00 | 0,000E+00 | 0,000E+00 | 3,137E+01 | 2,636E+02 | 0,000E+00 | 9,935E+00 | 0,000E+00 | 0,000E+00 | 0,000E+00 | 0,000E+00 | 0,000E+00 | 7,891E+01 | 0,000E+00 | 8,435E+01 | 2,937E+00 | 0,000E+00 | 0,000E+00 |
| Chim12 | 1,480E+02 | 3,180E+03 | 0,000E+00 | 1,065E+02 | 0,000E+00 | 0,000E+00 | 1,171E+01 | 0,000E+00 | 0,000E+00 | 0,000E+00 | 4,658E+02 | 0,000E+00 | 2,553E+02 | 0,000E+00 | 9,402E+01 | 7,452E+01 | 2,874E+02 | 1,514E+01 |
| Chim13 | 0,000E+00 | 0,000E+00 | 0,000E+00 | 0,000E+00 | 0,000E+00 | 0,000E+00 | 0,000E+00 | 0,000E+00 | 0,000E+00 | 0,000E+00 | 0,000E+00 | 0,000E+00 | 0,000E+00 | 0,000E+00 | 0,000E+00 | 0,000E+00 | 0,000E+00 | 0,000E+00 |
| Chim14 | 3,280E+01 | 0,000E+00 | 0,000E+00 | 1,523E+01 | 0,000E+00 | 9,918E+02 | 3,647E+00 | 0,000E+00 | 0,000E+00 | 0,000E+00 | 0,000E+00 | 0,000E+00 | 4,285E+01 | 0,000E+00 | 6,246E+01 | 1,213E+01 | 0,000E+00 | 1,926E+02 |
| ChiMo1 | 2,157E+01 | 0,000E+00 | 0,000E+00 | 4,466E+01 | 0,000E+00 | 5,158E+02 | 0,000E+00 | 0,000E+00 | 7,360E+04 | 0,000E+00 | 0,000E+00 | 0,000E+00 | 0,000E+00 | 0,000E+00 | 6,716E+01 | 1,656E+00 | 0,000E+00 | 1,295E+03 |
| ChiMo2 | 0,000E+00 | 0,000E+00 | 0,000E+00 | 1,189E+01 | 0,000E+00 | 1,636E+02 | 0,000E+00 | 0,000E+00 | 0,000E+00 | 0,000E+00 | 0,000E+00 | 0,000E+00 | 1,548E+01 | 0,000E+00 | 1,381E+01 | 0,000E+00 | 1,550E+02 | 5,364E+02 |
| ChiMo3 | 0,000E+00 | 0,000E+00 | 0,000E+00 | 6,267E+00 | 0,000E+00 | 0,000E+00 | 0,000E+00 | 0,000E+00 | 0,000E+00 | 0,000E+00 | 0,000E+00 | 0,000E+00 | 0,000E+00 | 0,000E+00 | 0,000E+00 | 0,000E+00 | 0,000E+00 | 0,000E+00 |
| ChiMo4 | 0,000E+00 | 0,000E+00 | 0,000E+00 | 5,675E+00 | 0,000E+00 | 0,000E+00 | 1,895E+01 | 0,000E+00 | 0,000E+00 | 0,000E+00 | 0,000E+00 | 0,000E+00 | 0,000E+00 | 0,000E+00 | 0,000E+00 | 0,000E+00 | 0,000E+00 | 0,000E+00 |
| ChiMo5 | 5,390E+01 | 1,674E+03 | 0,000E+00 | 1,442E+02 | 0,000E+00 | 3,911E+03 | 5,976E+02 | 0,000E+00 | 0,000E+00 | 0,000E+00 | 0,000E+00 | 0,000E+00 | 1,439E+03 | 4,591E+03 | 5,846E+02 | 1,596E+02 | 2,242E+03 | 3,236E+02 |
| ChiMo6-1 | 3,712E+00 | 0,000E+00 | 0,000E+00 | 4,402E+01 | 0,000E+00 | 0,000E+00 | 3,316E+00 | 0,000E+00 | 0,000E+00 | 0,000E+00 | 0,000E+00 | 0,000E+00 | 0,000E+00 | 0,000E+00 | 0,000E+00 | 2,168E+00 | 0,000E+00 | 0,000E+00 |
| ChiMo6-2 | 0,000E+00 | 0,000E+00 | 0,000E+00 | 4,629E+01 | 2,755E+02 | 7,217E+02 | 0,000E+00 | 0,000E+00 | 0,000E+00 | 0,000E+00 | 0,000E+00 | 0,000E+00 | 0,000E+00 | 0,000E+00 | 1,436E+02 | 2,776E+00 | 0,000E+00 | 0,000E+00 |
| ChiMo7 | 4,863E+00 | 0,000E+00 | 0,000E+00 | 1,496E+02 | 0,000E+00 | 3,592E+03 | 1,732E+01 | 0,000E+00 | 0,000E+00 | 0,000E+00 | 0,000E+00 | 0,000E+00 | 1,291E+02 | 0,000E+00 | 3,913E+01 | 1,204E+00 | 0,000E+00 | 0,000E+00 |
| ChiMo8 | 3,683E+01 | 0,000E+00 | 0,000E+00 | 2,303E+02 | 2,748E+03 | 4,280E+03 | 6,665E+01 | 0,000E+00 | 0,000E+00 | 1,257E+04 | 1,687E+03 | 0,000E+00 | 6,806E+02 | 9,687E+03 | 5,963E+02 | 4,543E+01 | 2,971E+02 | 8,561E+01 |
| ChiMo9 | 1,245E+01 | 9,659E+02 | 0,000E+00 | 2,396E+01 | 1,988E+02 | 0,000E+00 | 8,690E+00 | 0,000E+00 | 0,000E+00 | 0,000E+00 | 0,000E+00 | 0,000E+00 | 7,327E+01 | 0,000E+00 | 0,000E+00 | 7,339E+00 | 3,192E+02 | 0,000E+00 |
| ChiMo10 | 1,800E+01 | 0,000E+00 | 0,000E+00 | 7,362E+01 | 0,000E+00 | 1,461E+03 | 0,000E+00 | 0,000E+00 | 1,056E+05 | 0,000E+00 | 8,611E+02 | 0,000E+00 | 6,779E+02 | 5,790E+02 | 4,037E+02 | 3,947E+00 | 3,028E+02 | 1,119E+03 |
| ChiMo11 | 0,000E+00 | 0,000E+00 | 0,000E+00 | 0,000E+00 | 0,000E+00 | 0,000E+00 | 0,000E+00 | 0,000E+00 | 0,000E+00 | 0,000E+00 | 0,000E+00 | 0,000E+00 | 0,000E+00 | 0,000E+00 | 0,000E+00 | 0,000E+00 | 0,000E+00 | 0,000E+00 |

| **ENZYME** | | CORTISOL | | | *cis* -ANDROSTERONE | | | | NORANDROSTENEDIONE | | | | | | *trans* -ANDROSTERONE | | |
|---|---|---|---|---|---|---|---|---|---|---|---|---|---|---|---|---|---|
| | HC1 | HC2 | HC3 | cAD1 | cAD2 | cAD3 | cAD4 | NAD1 | NAD2 | NAD3 | NAD4 | NAD5 | NAD6 | tAD1 | tAD2 | tAD3 | tAD4 |
| Human wt CYP1A1-1 | 0,000E+00 | 0,000E+00 | 3,589E+02 | 1,749E+03 | 1,728E+03 | 1,073E+02 | 0,000E+00 | 1,695E+02 | 7,290E+02 | 0,000E+00 | 0,000E+00 | 1,137E+03 | 0,000E+00 | 0,000E+00 | 5,207E+03 | 0,000E+00 | 3,074E+02 |
| Human wt CYP1A1-2 | 0,000E+00 | 0,000E+00 | 2,476E+02 | 7,181E+02 | 3,090E+03 | 5,515E+01 | 0,000E+00 | 1,039E+02 | 2,456E+02 | 0,000E+00 | 0,000E+00 | 6,781E+02 | 0,000E+00 | 0,000E+00 | 1,836E+03 | 0,000E+00 | 2,348E+02 |
| Human wt CYP1A2-1 | 0,000E+00 | 0,000E+00 | 0,000E+00 | 0,000E+00 | 5,328E+03 | 4,726E+01 | 0,000E+00 | 2,494E+01 | 2,169E+02 | 6,245E+02 | 0,000E+00 | 0,000E+00 | 5,209E+03 | 0,000E+00 | 0,000E+00 | 0,000E+00 | 1,948E+02 |
| Human wt CYP1A2-2 | 0,000E+00 | 0,000E+00 | 0,000E+00 | 0,000E+00 | 2,410E+03 | 7,510E+00 | 0,000E+00 | 0,000E+00 | 0,000E+00 | 0,000E+00 | 0,000E+00 | 0,000E+00 | 6,119E+03 | 0,000E+00 | 0,000E+00 | 0,000E+00 | 9,520E+01 |
| Mouse wt CYP1A1-1 | 5,166E+03 | 0,000E+00 | 1,268E+02 | 1,300E+03 | 1,829E+03 | 4,898E+01 | 0,000E+00 | 6,660E+01 | 1,599E+02 | 0,000E+00 | 0,000E+00 | 1,152E+02 | 1,923E+03 | 0,000E+00 | 0,000E+00 | 0,000E+00 | 0,000E+00 |
| Mouse wt CYP1A1-2 | 0,000E+00 | 0,000E+00 | 5,604E+01 | 4,734E+02 | 0,000E+00 | 6,126E+01 | 0,000E+00 | 3,714E+01 | 1,015E+02 | 0,000E+00 | 0,000E+00 | 1,694E+02 | 0,000E+00 | 0,000E+00 | 0,000E+00 | 0,000E+00 | 1,001E+02 |
| Rabbit wt CYP1A2-1 | 4,295E+03 | 5,326E+04 | 2,625E+02 | 2,246E+03 | 2,628E+03 | 1,088E+02 | 2,837E+02 | 1,736E+02 | 3,437E+02 | 0,000E+00 | 0,000E+00 | 1,451E+03 | 4,303E+03 | 0,000E+00 | 1,091E+03 | 0,000E+00 | 5,866E+02 |
| Rabbit wt CYP1A2-2 | 1,413E+03 | 0,000E+00 | 5,112E+01 | 7,713E+02 | 5,792E+02 | 5,595E+01 | 1,419E+02 | 7,331E+01 | 1,690E+02 | 0,000E+00 | 0,000E+00 | 6,868E+02 | 3,804E+03 | 0,000E+00 | 3,084E+03 | 0,000E+00 | 3,123E+02 |
| Chim3 | 0,000E+00 | 0,000E+00 | 1,987E+02 | 1,379E+03 | 1,876E+03 | 9,472E+01 | 0,000E+00 | 7,959E+01 | 6,793E+02 | 0,000E+00 | 0,000E+00 | 0,000E+00 | 0,000E+00 | 8,173E+03 | 0,000E+00 | 0,000E+00 | 1,203E+02 |
| Chim4 | 0,000E+00 | 0,000E+00 | 1,540E+02 | 3,274E+03 | 1,410E+03 | 4,987E+01 | 0,000E+00 | 1,313E+02 | 2,191E+02 | 0,000E+00 | 0,000E+00 | 2,785E+02 | 0,000E+00 | 6,178E+03 | 3,094E+03 | 0,000E+00 | 6,776E+02 |
| Chim5 | 1,959E+04 | 0,000E+00 | 1,318E+03 | 4,737E+03 | 4,970E+03 | 1,134E+02 | 0,000E+00 | 5,353E+02 | 7,565E+02 | 0,000E+00 | 0,000E+00 | 3,651E+03 | 0,000E+00 | 0,000E+00 | 1,231E+03 | 0,000E+00 | 1,367E+03 |
| Chim6 | 2,847E+03 | 0,000E+00 | 4,277E+02 | 3,679E+03 | 2,117E+03 | 1,036E+02 | 0,000E+00 | 1,272E+02 | 4,058E+02 | 0,000E+00 | 0,000E+00 | 3,922E+02 | 0,000E+00 | 1,226E+04 | 1,894E+03 | 0,000E+00 | 1,202E+03 |
| Chim7 | 0,000E+00 | 0,000E+00 | 1,811E+02 | 8,752E+02 | 0,000E+00 | 7,626E+01 | 0,000E+00 | 1,356E+02 | 1,572E+02 | 7,127E+01 | 4,216E+04 | 1,237E+03 | 0,000E+00 | 3,904E+03 | 0,000E+00 | 0,000E+00 | 5,535E+02 |
| Chim8 | 0,000E+00 | 0,000E+00 | 9,411E+01 | 0,000E+00 | 0,000E+00 | 0,000E+00 | 0,000E+00 | 6,021E+01 | 1,853E+02 | 0,000E+00 | 0,000E+00 | 6,873E+02 | 0,000E+00 | 0,000E+00 | 0,000E+00 | 0,000E+00 | 0,000E+00 |
| Chim9 | 3,450E+03 | 0,000E+00 | 2,206E+02 | 1,408E+03 | 1,878E+03 | 1,118E+02 | 0,000E+00 | 8,287E+01 | 2,172E+02 | 0,000E+00 | 0,000E+00 | 5,429E+02 | 0,000E+00 | 0,000E+00 | 8,768E+02 | 0,000E+00 | 3,207E+02 |
| Chim11 | 0,000E+00 | 0,000E+00 | 2,811E+02 | 2,062E+02 | 1,123E+03 | 2,403E+01 | 0,000E+00 | 3,512E+01 | 1,785E+02 | 0,000E+00 | 0,000E+00 | 2,024E+02 | 0,000E+00 | 6,547E+03 | 0,000E+00 | 0,000E+00 | 7,480E+01 |
| Chim12 | 2,847E+03 | 0,000E+00 | 4,277E+02 | 3,679E+03 | 2,117E+03 | 1,036E+02 | 0,000E+00 | 1,272E+02 | 4,058E+02 | 0,000E+00 | 0,000E+00 | 3,922E+02 | 0,000E+00 | 1,226E+04 | 1,894E+03 | 0,000E+00 | 1,202E+03 |
| Chim13 | 0,000E+00 | 0,000E+00 | 0,000E+00 | 0,000E+00 | 0,000E+00 | 0,000E+00 | 0,000E+00 | 0,000E+00 | 0,000E+00 | 0,000E+00 | 0,000E+00 | 0,000E+00 | 0,000E+00 | 0,000E+00 | 0,000E+00 | 0,000E+00 | 0,000E+00 |
| Chim14 | 0,000E+00 | 0,000E+00 | 2,804E+01 | 3,545E+02 | 0,000E+00 | 4,209E+01 | 0,000E+00 | 4,309E+01 | 1,874E+02 | 0,000E+00 | 0,000E+00 | 8,575E+02 | 0,000E+00 | 0,000E+00 | 1,520E+03 | 0,000E+00 | 1,868E+02 |
| ChiMo1 | 0,000E+00 | 0,000E+00 | 7,652E+01 | 0,000E+00 | 8,855E+02 | 1,996E+02 | 0,000E+00 | 8,008E+01 | 1,808E+02 | 2,392E+02 | 0,000E+00 | 3,239E+02 | 2,580E+03 | 1,699E+04 | 0,000E+00 | 0,000E+00 | 2,704E+02 |
| ChiMo2 | 0,000E+00 | 0,000E+00 | 0,000E+00 | 0,000E+00 | 0,000E+00 | 4,585E+02 | 0,000E+00 | 2,140E+01 | 1,393E+02 | 2,219E+02 | 0,000E+00 | 0,000E+00 | 0,000E+00 | 1,491E+04 | 0,000E+00 | 0,000E+00 | 5,290E+01 |
| ChiMo3 | 0,000E+00 | 0,000E+00 | 3,503E+01 | 0,000E+00 | 0,000E+00 | 1,050E+02 | 0,000E+00 | 0,000E+00 | 0,000E+00 | 2,318E+01 | 0,000E+00 | 0,000E+00 | 0,000E+00 | 0,000E+00 | 0,000E+00 | 0,000E+00 | 0,000E+00 |
| ChiMo4 | 0,000E+00 | 0,000E+00 | 0,000E+00 | 0,000E+00 | 0,000E+00 | 1,817E+02 | 0,000E+00 | 0,000E+00 | 4,209E+01 | 0,000E+00 | 0,000E+00 | 0,000E+00 | 0,000E+00 | 0,000E+00 | 0,000E+00 | 0,000E+00 | 0,000E+00 |
| ChiMo5 | 0,000E+00 | 0,000E+00 | 5,056E+02 | 5,984E+03 | 7,486E+03 | 3,645E+02 | 0,000E+00 | 2,353E+02 | 7,355E+02 | 0,000E+00 | 0,000E+00 | 2,085E+03 | 4,739E+03 | 0,000E+00 | 5,302E+03 | 0,000E+00 | 6,645E+02 |
| ChiMo6-1 | 0,000E+00 | 0,000E+00 | 4,142E+02 | 0,000E+00 | 0,000E+00 | 9,059E+01 | 7,311E+02 | 0,000E+00 | 0,000E+00 | 0,000E+00 | 0,000E+00 | 0,000E+00 | 0,000E+00 | 0,000E+00 | 0,000E+00 | 0,000E+00 | 5,872E+01 |
| ChiMo6-2 | 0,000E+00 | 0,000E+00 | 0,000E+00 | 0,000E+00 | 0,000E+00 | 4,561E+01 | 0,000E+00 | 3,208E+01 | 8,393E+01 | 0,000E+00 | 0,000E+00 | 0,000E+00 | 0,000E+00 | 0,000E+00 | 0,000E+00 | 0,000E+00 | 0,000E+00 |
| ChiMo7 | 0,000E+00 | 0,000E+00 | 5,774E+02 | 1,942E+02 | 0,000E+00 | 6,357E+02 | 4,426E+03 | 6,044E+01 | 1,840E+02 | 0,000E+00 | 0,000E+00 | 6,378E+02 | 0,000E+00 | 0,000E+00 | 7,697E+02 | 0,000E+00 | 1,068E+02 |
| ChiMo8 | 1,524E+03 | 0,000E+00 | 5,209E+02 | 6,131E+02 | 2,585E+03 | 1,847E+02 | 2,621E+02 | 4,034E+02 | 7,195E+02 | 6,309E+01 | 0,000E+00 | 3,026E+03 | 9,886E+03 | 1,026E+04 | 1,132E+03 | 0,000E+00 | 1,149E+03 |
| ChiMo9 | 0,000E+00 | 0,000E+00 | 5,928E+01 | 0,000E+00 | 0,000E+00 | 2,978E+02 | 1,083E+03 | 6,729E+01 | 2,737E+02 | 0,000E+00 | 0,000E+00 | 1,720E+02 | 0,000E+00 | 2,180E+04 | 0,000E+00 | 0,000E+00 | 4,919E+01 |
| ChiMo10 | 0,000E+00 | 0,000E+00 | 0,000E+00 | 7,710E+02 | 0,000E+00 | 6,197E+01 | 0,000E+00 | 1,821E+02 | 2,567E+02 | 1,835E+03 | 0,000E+00 | 7,700E+02 | 0,000E+00 | 0,000E+00 | 5,733E+03 | 0,000E+00 | 9,504E+02 |
| ChiMo11 | 0,000E+00 | 0,000E+00 | 0,000E+00 | 0,000E+00 | 0,000E+00 | 0,000E+00 | 0,000E+00 | 0,000E+00 | 0,000E+00 | 0,000E+00 | 0,000E+00 | 0,000E+00 | 0,000E+00 | 0,000E+00 | 0,000E+00 | 0,000E+00 | 0,000E+00 |
